# Author Correction: Residual apoptotic activity of a tumorigenic p53 mutant improves cancer therapy responses

**DOI:** 10.1038/s44318-026-00758-4

**Published:** 2026-03-24

**Authors:** Oleg Timofeev, Boris Klimovich, Jean Schneikert, Michael Wanzel, Evangelos Pavlakis, Julia Noll, Samet Mutlu, Sabrina Elmshäuser, Andrea Nist, Marco Mernberger, Boris Lamp, Ulrich Wenig, Alexander Brobeil, Stefan Gattenlöhner, Kernt Köhler, Thorsten Stiewe

**Affiliations:** 1 Institute of Molecular Oncology, Philipps-University, Marburg, Germany; 2 Universities of Giessen and Marburg Lung Center, German Center for Lung Research (DZL), Marburg, Germany; 3 Genomics Core Facility, Philipps University, Marburg, Germany; 4 Institute of Pathology, Justus Liebig University, Giessen, Germany; 5 Institute of Veterinary Pathology, Justus Liebig University, Giessen, Germany

## Abstract

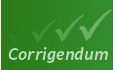

**Correction to:** *The EMBO Journal* (2019) 38:e102096. 10.15252/embj.2019102096 | Published online 4 September 2019

The authors contacted the journal after being made aware of a possible reuse within Figure 7G. The authors supplied the journal with direct exports of the unmodified raw data. The journal verified the raw data and agrees to retract and replace Figure 7G.

**Figure 7G is corrected**.

Author statement: We were made aware of a reuse of image in the untreated Trp53^+^/^+^ cohort at Day 0. This duplication resulted from a copy–paste error during figure assembly. We have carefully re-examined the original source data and confirmed that this error is confined to this panel and does not affect any other figures presented in the manuscript.

We have provided a corrected version of Figure 7G.

**Figure 7:**
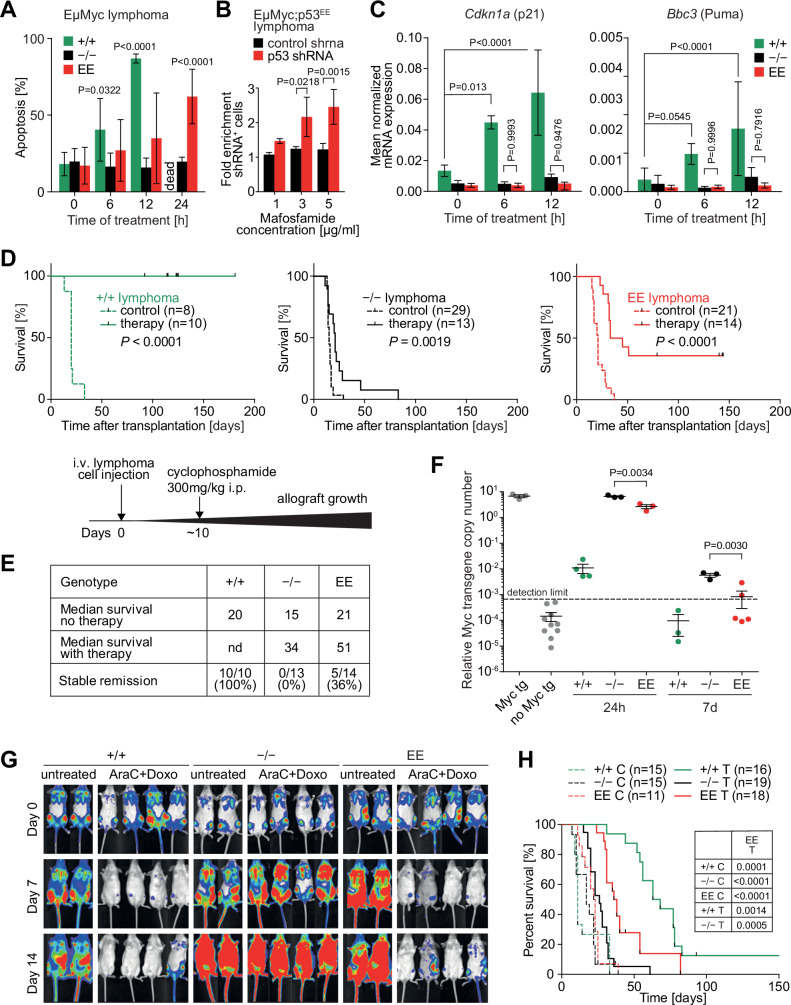
**Original**.

**Figure 7:**
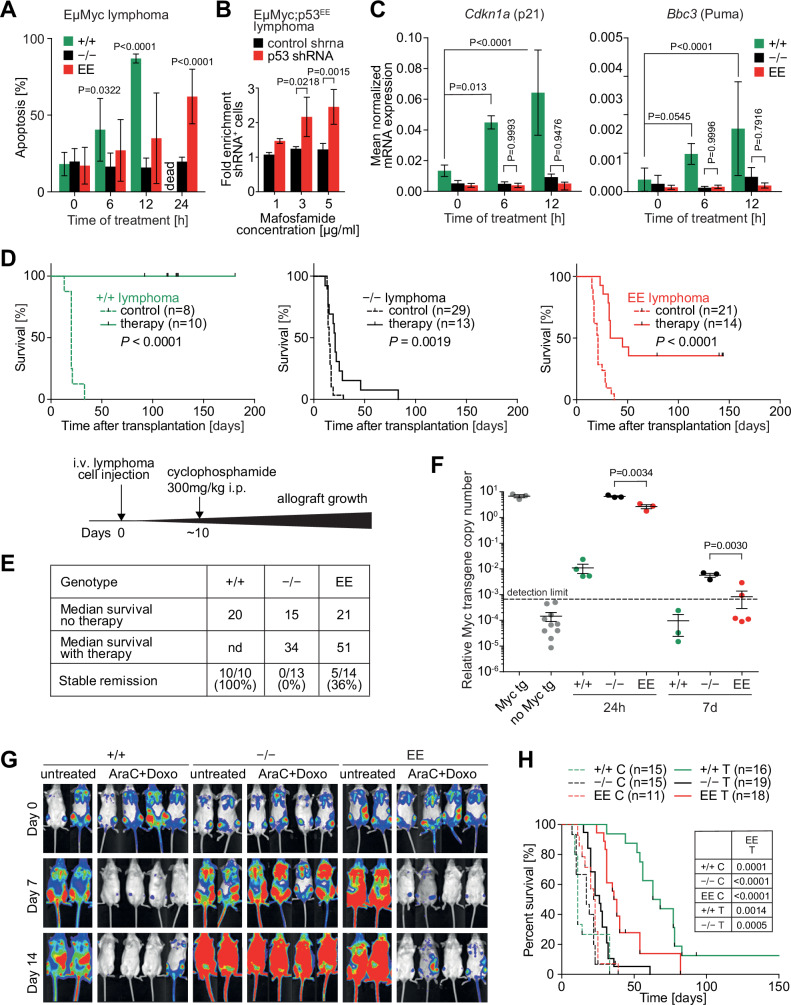
**Corrected**. Source data are available online for this figure.

All authors agree to this correction and apologise for any inconvenience caused.

## Supplementary information

Figure 7G Source Data

