# Supplementary material for: Residual apoptotic activity of a tumorigenic p53 mutant improves cancer therapy responses
Source: EMBO J. 2019 Sep 4;38(20):e102096. doi: 10.15252/embj.2019102096 (PMC6792016; doi:10.15252/embj.2019102096)
Supplement: Supplementary file 1 — Appendix [file EMBJ-38-e102096-s001.pdf]

Appendix

Residual apoptotic activity of a tumorigenic p53 mutant improves cancer therapy responses

Appendix tables

Table S1. Histopathological analysis of tumors from $p53^{EE/EE}$, $p53^{–/EE}$ and $p53^{–/–}$ mice.

Table S2. Histopathological analysis of tumors from $p53^{+/–}$ and $p53^{+/EE}$ mice.

Appendix Table S1. Histopathological analysis of tumors from $p53^{EE/EE}$, $p53^{–/EE}$ and $p53^{–/–}$ mice.

Sample No	*Trp53* genotype	Age	Diagnosis
1	–/–	59	Sarcoma
2	–/–	186	Thymic lymphoma
3	–/–	215	B-cell lymphoma
4	–/–	128	Thymic lymphoma
5	–/–	129	Thymic lymphoma
6	–/–	155	Thymic lymphoma
7	–/–	143	Thymic lymphoma
8	–/–	89	Sarcoma
9	–/–	186	Thymic lymphoma
10	–/–	186	Hemangiosarcoma
11	–/–	101	Thymic lymphoma
12	–/–	110	Thymic lymphoma
13	–/–	119	Thymic lymphoma
14	–/–	141	Thymic lymphoma
15	–/–	154	Thymic lymphoma
16	–/–	226	Thymic lymphoma
1	–/EE	85	Thymic lymphoma
2	–/EE	112	Thymic lymphoma
3	–/EE	123	Thymic lymphoma
4	–/EE	120	Thymic lymphoma
5	–/EE	114	Thymic lymphoma
6	–/EE	164	Thymic lymphoma
7	–/EE	204	Thymic lymphoma
8	–/EE	167	Thymic lymphoma
9	–/EE	91	Non-malignant Sertoli cell tumor
10	–/EE	100	Thymic lymphoma
11	–/EE	177	Thymic lymphoma
12	–/EE	99	Thymic lymphoma
13	–/EE	203	Thymic lymphoma
14	–/EE	135	Thymic lymphoma
15	–/EE	139	Malignant teratoma
16	–/EE	214	(Spleen: lymphatic hyperplasia)
17	–/EE	78	Sarcoma
18	–/EE	107	Thymic lymphoma
19	–/EE	128	Thymic lymphoma
20	–/EE	184	Thymic lymphoma
21	–/EE	114	Spindle cell sarcoma
22	–/EE	114	Malignant teratoma
23	–/EE	116	Thymic lymphoma
24	–/EE	120	Thymic lymphoma
25	–/EE	212	Hemangiosarcoma
26	–/EE	168	B-cell lymphoma
1	EE/EE	110	Thymic lymphoma

2	EE/EE	125	Thymic lymphoma
3	EE/EE	150	Thymic lymphoma
4	EE/EE	35	B-lymphoma
5	EE/EE	151	B-lymphoma
6	EE/EE	101	Carcinoma
7	EE/EE	74	B-lymphoma
8	EE/EE	192	B-lymphoma
9	EE/EE	258	**Metastatic spindle cell sarcoma**
10	EE/EE	157	Thymic lymphoma
11	EE/EE	124	Thymic lymphoma
12	EE/EE	213	Thymic lymphoma
13	EE/EE	213	Malignant seminoma
14	EE/EE	142	Thymic lymphoma
15	EE/EE	34	Thymic lymphoma
16	EE/EE	146	Thymic lymphoma
17	EE/EE	81	B-lymphoma
18	EE/EE	88	(Spleen: lymphatic hyperplasia)
19	EE/EE	133	Thymic lymphoma
20	EE/EE	262	Thymic lymphoma
21	EE/EE	262	**Metastatic osteosarcoma**
22	EE/EE	158	Thymic lymphoma
23	EE/EE	154	Thymic lymphoma
24	EE/EE	192	Thymic lymphoma
25	EE/EE	130	Thymic lymphoma
26	EE/EE	154	Thymic lymphoma
27	EE/EE	208	Thymic lymphoma
28	EE/EE	159	Thymic lymphoma
29	EE/EE	291	Hemangiosarcoma
30	EE/EE	150	Thymic lymphoma
31	EE/EE	223	Thymic lymphoma
32	EE/EE	189	Thymic lymphoma
33	EE/EE	183	Thymic lymphoma
34	EE/EE	304	Thymic lymphoma
35	EE/EE	265	Thymic lymphoma
36	EE/EE	298	B-lymphoma
37	EE/EE	112	(Spleen: lymphatic hyperplasia)
38	EE/EE	119	Thymic lymphoma
39	EE/EE	114	Thymic lymphoma

Appendix Table S2. Histopathological analysis of tumors from $p53^{+/–}$ and $p53^{+/EE}$ mice. Metastatic tumors are listed in bold, non-malignant tumors in parentheses.

Sample No	*Trp53* genotype	Age	Diagnosis
1	+/–	242	Lymphoma
2	+/–	400	Tubulo-papillary carcinoma
3	+/–	439	Osteosarcoma
4	+/–	511	Solid carcinoma
5	+/–	480	Tubulo-papillary carcinoma
6	+/–	412	Thymic lymphoma
7	+/–	412	**Metastatic mesothelioma or carcinoma**
8	+/–	514	(Spleen: lymphatic hyperplasia)
9	+/–	601	Lymphoma
10	+/–	601	**Metastatic osteoplastic sarcoma**
11	+/–	542	Tubulo-papillary carcinoma
12	+/–	534	Keratinizing squamous cell carcinoma
13	+/–	421	Lymphoma
14	+/–	651	Lymphoma
15	+/–	611	Lymphoma
16	+/–	611	Fibrosarcoma
17	+/–	449	Lymphoma
18	+/–	578	Keratinizing squamous cell carcinoma
19	+/–	520	Sarcoma
20	+/–	647	Sarcoma
21	+/–	703	Nephroblastoma
22	+/–	636	Hemangiosarcoma
23	+/–	668	Osteosarcoma
24	+/–	612	(Non-malignant hemangioma)
25	+/–	598	**Malignant metastatic blastoma**
26	+/–	625	Lymphoma
27	+/–	625	Hemangiosarcoma
28	+/–	616	Sarcoma
29	+/–	616	Bronchial carcinoma
30	+/–	621	Sarcoma
31	+/–	621	Bronchial carcinoma
32	+/–	425	Lymphoma
33	+/–	412	Lymphoma
1	+/EE	341	Lymphoma
2	+/EE	250	Lymphoma
3	+/EE	295	(Non-malignant hemangioma)
4	+/EE	391	Lymphoma
5	+/EE	425	Tubulo-papillary carcinoma
6	+/EE	277	(Non-malignant hemangioma)
7	+/EE	481	Lymphoma
8	+/EE	579	**Metastatic osteosarcoma**
9	+/EE	514	Malignant blastoma
10	+/EE	413	Malignant blastoma
11	+/EE	443	Malignant blastoma

12	+/EE	519	(Spleen: lymphatic hyperplasia)
13	+/EE	448	Sarcoma
14	+/EE	637	**Metastatic osteosarcoma**
15	+/EE	583	Endometrium: hyperplasia
16	+/EE	570	Malignant teratoma
17	+/EE	681	Hemangiosarcoma
18	+/EE	567	Malignant granulosa cell tumor
19	+/EE	760	Lymphoma
20	+/EE	591	(Spleen: lymphatic hyperplasia)
21	+/EE	631	**Metastatic hemangiosarcoma**
22	+/EE	676	**Metastatic bronchial carcinoma**
