# Supplementary material for: Residual apoptotic activity of a tumorigenic p53 mutant improves cancer therapy responses
Source: EMBO J. 2019 Sep 4;38(20):e102096. doi: 10.15252/embj.2019102096 (PMC6792016; doi:10.15252/embj.2019102096)
Supplement: Supplementary file 2 — Expanded View Figures PDF [file EMBJ-38-e102096-s002.pdf]

Expanded View Figures

Figure EV1. Generation and characterization of the *Trp53*^{R178E} knock-in mouse.

A *Trp53* targeting strategy. Asterisk indicates the R178E (EE) point mutation in exon 5; LSL, lox-stop-lox cassette.
B Southern blot, showing integration of the construct in a correctly targeted 129/SvEv embryonic stem cell clone. Genomic DNA was digested with *Ssp*I and hybridized with the 3′ probe shown in (A). LSL-EE denotes the targeted allele carrying a lox-stop-lox (LSL) cassette and R178E (EE) mutation. The 10.3 kb *Ssp*I fragment corresponds to the wild-type and the 8.4 kb fragment to the targeted allele.
C Sanger sequencing of a *Trp53* exon 5-6 PCR amplicon confirms the presence of the Arg->Glu mutation in a tiptail biopsy from a heterozygous founder mouse.
D PCR used for genotyping of mouse biopsies and cells. Asterisk indicates unspecific PCR product.
E Electrophoretic mobility shift assay (EMSA) performed with a radiolabeled oligonucleotide containing a p53 consensus binding site incubated with *in vitro* translated full-length p53 protein (IVT p53WT, left) or nuclear extracts from primary MEFs with indicated p53 genotypes treated with 10 μM Nutlin o/n (right). For supershift analysis, anti-p53 antibody (FL-393, Santa Cruz) was added; asterisks denote disrupted and shifted bands, respectively. Arrowhead, specific p53-DNA complex. ret lys—reticulocyte lysate; specific comp—non-radiolabeled consensus binding site oligonucleotide as competitor; scrambled comp—non-radiolabeled sequence-scrambled competitor oligonucleotide; ns—non-specific.
F Venn diagram illustrating number and overlap of peaks called in the p53 ChIP-seq datasets from Nutlin-treated MEFs of indicated genotypes. Only peaks present in p53$^{+/+}$—but not in p53$^{-/-}$ MEFs—were considered p53-specific.
G Hypergeometric enrichment showing that genes in the vicinity of p53 ChIP-seq peaks from Nutlin-treated p53$^{+/+}$ MEFs are significantly enriched for p53-related gene sets from the Molecular Signatures Database (MSigDB). Shown is the -log10 of the *P* value adjusted for multiple comparisons using Benjamini–Hochberg correction.
H Nutlin-regulated gene expression in primary MEFs of indicated p53 genotypes. Scatter plot shows the log2-fold change of the 1,000 top-regulated genes. Box and whiskers indicate the interquartile range and 5–95 percentiles, respectively. Significance was tested by ordinary ANOVA with Sidak's multiple comparisons test.
I p53EE fails to regulate non-canonical tumor-suppressive target genes identified by transcriptional profiling of transactivation domain mutant mice (Brady *et al*, 2011). Shown are the z-transformed RNA expression values (FPKM).

© 2019 The Authors

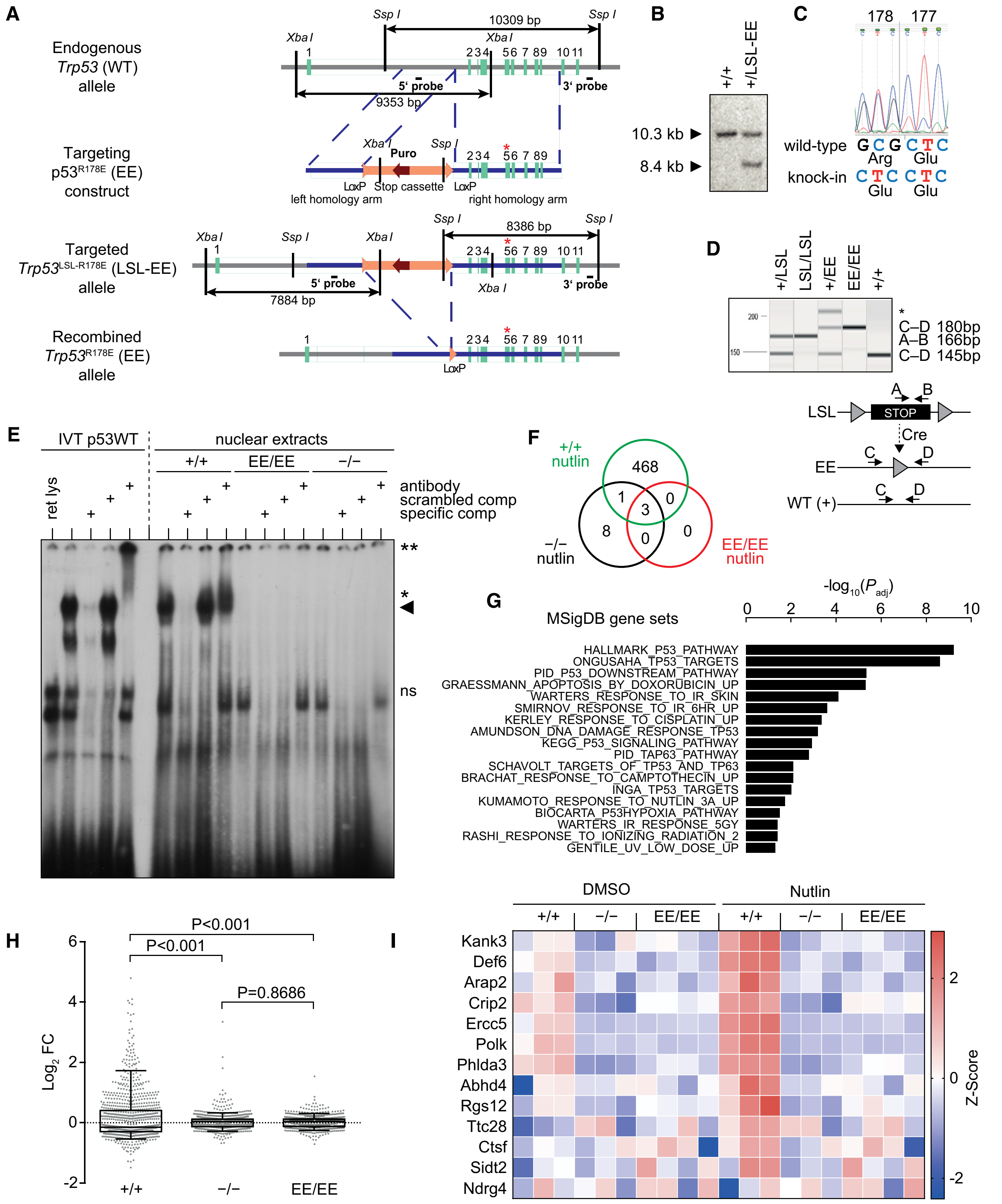
A
Endogenous Trp53 (WT) allele
Targeting p53R178E (EE) construct
Targeted Trp53LSL-R178E (LSL-EE) allele
Recombined Trp53R178E (EE) allele
Ssp I
Xba I
10309 bp
9353 bp
8386 bp
7884 bp
5' probe
3' probe
Puro
LoxP
Stop cassette
left homology arm
right homology arm
B
+/+
+/LSL-EE
10.3 kb
8.4 kb
C
178
177
wild-type
knock-in
Arg
Glu
D
+/LSL
LSL/LSL
+/EE
EE/EE
+/+
C–D 180bp
A–B 166bp
C–D 145bp
LSL
STOP
Cre
EE
WT (+)
E
IVT p53WT
nuclear extracts
+/+
EE/EE
−/−
ret lys
antibody
scrambled comp
specific comp
ns
F
+/+ nutlin
−/− nutlin
EE/EE nutlin
G
MSigDB gene sets
-log10(Padj)
HALLMARK_P53_PATHWAY
ONGUSAHA_TP53_TARGETS
PID_P53_DOWNSTREAM_PATHWAY
GRAESSMANN_APOPTOSIS_BY_DOXORUBICIN_UP
WARTERS_RESPONSE_TO_IR_SKIN
SMIRNOV_RESPONSE_TO_IR_6HR_UP
KERLEY_RESPONSE_TO_CISPLATIN_UP
AMUNDSON_DNA_DAMAGE_RESPONSE_TP53
KEGG_P53_SIGNALING_PATHWAY
PID_TAP63_PATHWAY
SCHAVOLT_TARGETS_OF_TP53_AND_TP63
BRACHAT_RESPONSE_TO_CAMPTOTHECIN_UP
INGA_TP53_TARGETS
KUMAMOTO_RESPONSE_TO_NUTLIN_3A_UP
BIOCARTA_P53HYPOXIA_PATHWAY
WARTERS_IR_RESPONSE_5GY
RASHI_RESPONSE_TO_IONIZING_RADIATION_2
GENTILE_UV_LOW_DOSE_UP
H
P<0.001
P<0.001
P=0.8686
Log2 FC
+/+
−/−
EE/EE
I
DMSO
Nutlin
Kank3
Def6
Arap2
Crip2
Ercc5
Polk
Phlda3
Abhd4
Rgs12
Ttc28
Ctsf
Sidt2
Ndrg4
Z-Score

Figure EV1.

Figure EV2. Cells and tissues from p53EE mice show deficiency in cell cycle arrest, senescence, and apoptosis.

A Oncogenic $HRas^{G12V}$ was overexpressed in primary MEFs, and cells were stained for senescence-associated β-galactosidase (SA β-gal) to detect oncogene-induced senescence (positive cells are marked with arrowheads). Western blots show expression of H-Ras and β-actin as loading control.

B Senescence caused by cell culture stress was assessed in primary MEFs at passage 8 by SA β-gal staining (arrowheads).

C *Left*, 3D structure of the double salt bridge formed between the H1 helices of two adjacent p53WT monomers. Residues glutamate 177 (177E) and arginine 178 (178R) are labeled. *Right*, schematic illustration of interactions between the two H1 helices of p53 molecules with wild-type and mutated Glu177 and Arg178.

D Primary thymocytes were isolated from mice with indicated genotypes and irradiated *ex vivo* with 6 Gy X-ray. Cellular survival at indicated time points was analyzed using CellTiter-Glo assay (Promega). Note, homozygous EE and homozygous RR mutant thymocytes are apoptosis-deficient, while compound EE/RR mutant thymocytes are sensitive to irradiation. Data are shown as mean ± SD.

E Apoptosis (detected by IHC staining of cleaved caspase-3) in thymus of control or irradiated mice (6 Gy X-ray) after 6 h. Note massive apoptosis in both $p53^{+/+}$ and $p53^{EE/RR}$ mice.

F The absence of p53EE expression in small intestine of unstressed mice and accumulation at indicated time points after irradiation. $p53^{+/+}$ are shown for comparison.

G Dynamics of apoptosis (TUNEL staining) in small intestine at indicated time points after whole-body irradiation (6 Gy X-ray). Arrowheads mark TUNEL-positive apoptotic cells.

H Cell proliferation in samples from (G) as detected by immunohistochemical staining for BrdU incorporation.

Data information: All scale bars denote 50 μm.

 © 2019 The Authors

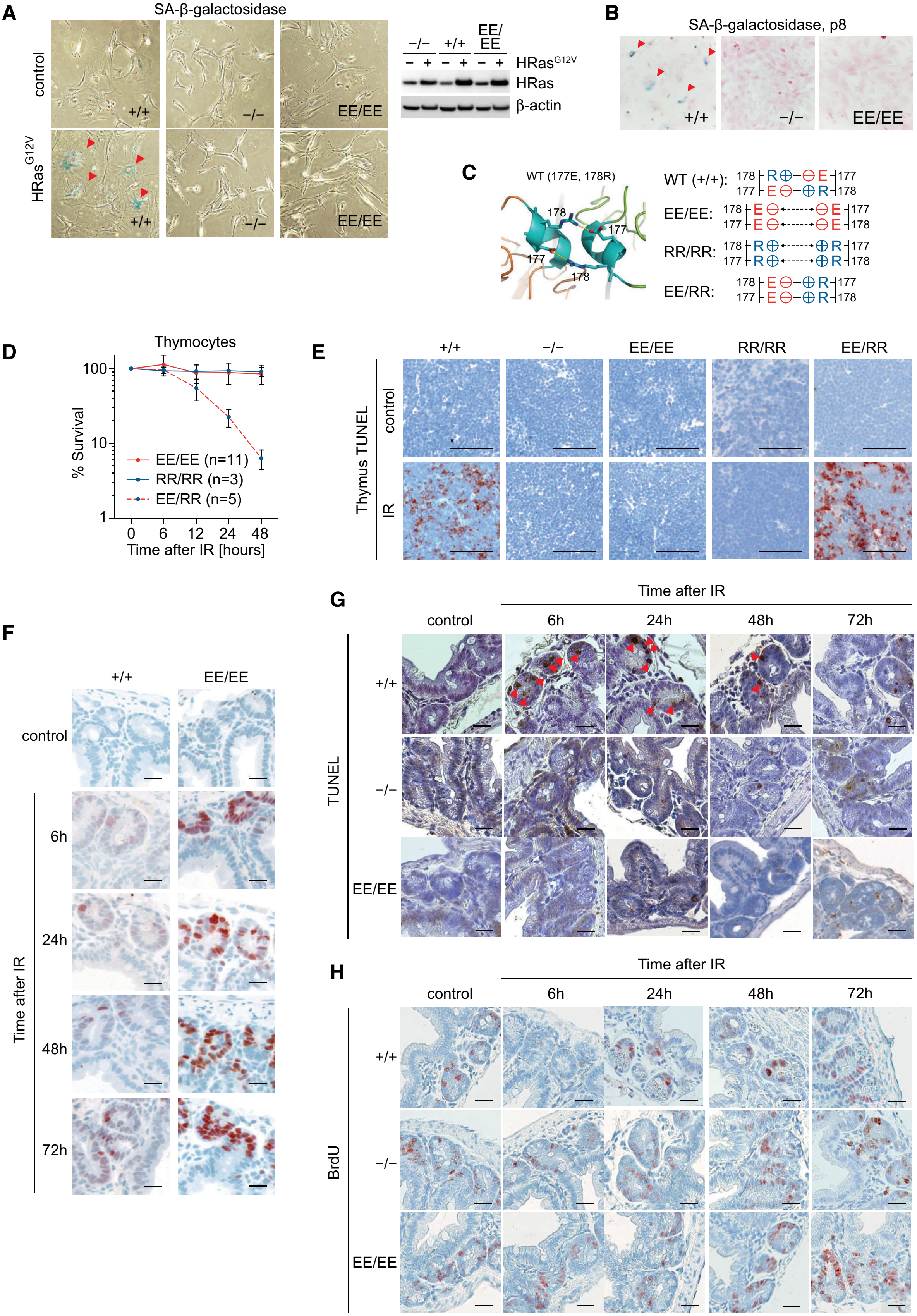
A
SA-β-galactosidase
control
HRasG12V
+/+
−/−
EE/EE
−/− +/+ EE/EE
HRasG12V
HRas
β-actin
B
SA-β-galactosidase, p8
+/+
−/−
EE/EE
C
WT (177E, 178R)
WT (+/+):
EE/EE:
RR/RR:
EE/RR:
D
Thymocytes
% Survival
Time after IR [hours]
EE/EE (n=11)
RR/RR (n=3)
EE/RR (n=5)
E
+/+
−/−
EE/EE
RR/RR
EE/RR
Thymus TUNEL
control
IR
F
control
6h
24h
48h
72h
Time after IR
G
Time after IR
control
6h
24h
48h
72h
TUNEL
H
BrdU

Figure EV2.

Figure EV3. Constitutive p53EE stabilization triggers ROS-dependent senescence.

A Long-term proliferation assay for freshly isolated primary MEFs with the genotypes $p53^{-/-}$ (n = 3), $p53^{+/+}$ (n = 3), and $p53^{EE/EE}$ (n = 6).

B SA-β-galactosidase staining of MEFs from (A) at passage 16. Red arrowheads indicate senescent cells positive for SA-β-galactosidase.

C Western blot of MEFs with indicated genotypes at early (p3) and late passages ($p53^{+/+}$ p7, $p53^{-/-}$, and $p53^{EE/EE}$ p17). Asterisk marks a spontaneously immortalized $p53^{EE/EE}$ cell line, which has lost p53 expression. s.e., short exposure; l.e., long exposure.

D mRNA expression analysis (RT–qPCR) of MEF cultures from (C). mRNA expression was normalized to β-actin.

E Long-term proliferation assay with primary $p53^{-/-}$ and $p53^{EE/EE}$ MEFs transduced with two different CRISPR/Cas9 nucleases targeting *Trp53* or GFP (control). Shown is the passage number after gene editing.

F Mitochondrial ROS in primary MEFs from late passages measured by flow cytometry with MitoSOX red dye. EE*, spontaneously immortalized $p53^{EE/EE}$ cell culture with loss of p53 expression.

G mRNA expression analysis (RT–qPCR) of early and late passage MEFs cultured in low (3%) oxygen; n = 4. mRNA expression was normalized to β-actin.

H Long-term proliferation of primary $p53^{-/-}$ and $p53^{EE/EE}$ MEFs cultured from frozen stocks in normal high (21%) versus low (3%) oxygen. Shown is the number of passages after revitalization of MEFs frozen at passage 2–4. n = 4.

I Oxygen consumption rate (OCR) assessed with Seahorse XF Cell Mito Stress Test Kit. pInd20-p53EE, Tet-induced p53EE expression in *Trp53*$^{-/-}$;*Mdm2*$^{-/-}$ MEFs. Control, Tet-treated *Trp53*$^{-/-}$;*Mdm2*$^{-/-}$ MEFs. Time points of treatment with oligomycin, FCCP, and rotenone+antimycin A are indicated with arrows. Statistical significance was tested with multiple two-sided t-tests in combination with the false discovery rate approach. FDR q-values < 0.05 are considered significant (n = 8).

J Mitochondrial DNA (mtDNA) content of indicated MEFs determined by qPCR and normalized to early passage $p53^{+/+}$ MEFs and nuclear DNA (nuDNA). $p53^{+/+}$ early, n = 3; $p53^{+/+}$ late, n = 4; $p53^{-/-}$ early, n = 1; $p53^{-/-}$ late, n = 1; $p53^{EE/EE}$ early, n = 3; $p53^{EE/EE}$ late, n = 6.

K mRNA expression analysis (RT–qPCR) of $p53^{-/-}$ and $p53^{EE/EE}$ MEF cultures for indicated Nrf2 target genes. mRNA expression was normalized to β-actin; n = 3.

L *Hmox1* mRNA expression analysis (RT–qPCR) of $p53^{-/-}$ and $p53^{EE/EE}$ MEF cultures treated for 4 h with 0, 50, 100, 200, 400, and 800 μM H_2O_2. mRNA expression was normalized to β-actin; n = 3.

Data information: All data are shown as mean ± SD. Significance was tested by 2-way ANOVA with Sidak's multiple comparisons test unless indicated otherwise.

 © 2019 The Authors

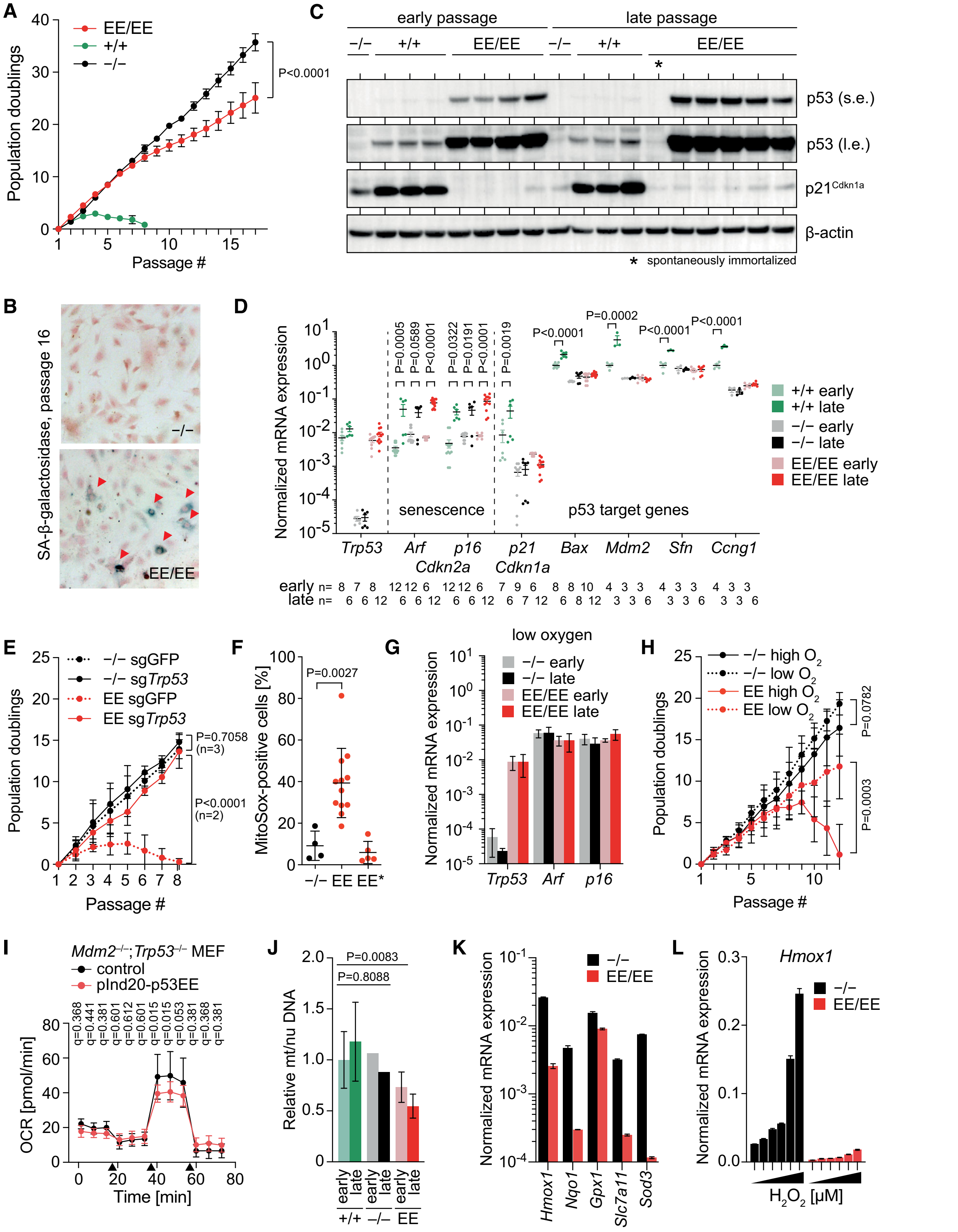
A
Population doublings
Passage #
EE/EE
+/+
−/−
P<0.0001
C
early passage
late passage
−/− +/+ EE/EE −/− +/+ EE/EE
p53 (s.e.)
p53 (l.e.)
p21Cdkn1a
β-actin
* spontaneously immortalized
B
SA-β-galactosidase, passage 16
−/−
EE/EE
D
Normalized mRNA expression
senescence
p53 target genes
Trp53 Arf Cdkn2a p16 p21 Cdkn1a Bax Mdm2 Sfn Ccng1
P=0.0005 P=0.0589 P<0.0001 P=0.0322 P=0.0191 P<0.0001 P=0.0019
P<0.0001 P=0.0002 P<0.0001 P<0.0001
+/+ early
+/+ late
−/− early
−/− late
EE/EE early
EE/EE late
early n= 8 7 8 12 12 6 12 12 6 7 9 6 8 8 10 4 3 3 4 3 3 4 3 3
late n= 6 6 12 6 6 12 6 6 12 6 7 12 6 8 12 3 3 6 3 3 6 3 3 6
E
Population doublings
Passage #
−/− sgGFP
−/− sgTrp53
EE sgGFP
EE sgTrp53
P=0.7058 (n=3)
P<0.0001 (n=2)
F
MitoSox-positive cells [%]
P=0.0027
−/− EE EE*
G
low oxygen
Normalized mRNA expression
−/− early
−/− late
EE/EE early
EE/EE late
Trp53 Arf p16
H
Population doublings
Passage #
−/− high O2
−/− low O2
EE high O2
EE low O2
P=0.0782
P=0.0003
I
Mdm2−/−;Trp53−/− MEF
control
pInd20-p53EE
q=0.368 q=0.441 q=0.381 q=0.601 q=0.612 q=0.601 q=0.015 q=0.015 q=0.053 q=0.381 q=0.368 q=0.381
OCR [pmol/min]
Time [min]
J
Relative mt/nu DNA
P=0.0083
P=0.8088
early late early late early late
+/+ −/− EE
K
Normalized mRNA expression
−/−
EE/EE
Hmox1 Nqo1 Gpx1 Slc7a11 Sod3
L
Hmox1
Normalized mRNA expression
−/−
EE/EE
H2O2 [μM]

Figure EV3.

A

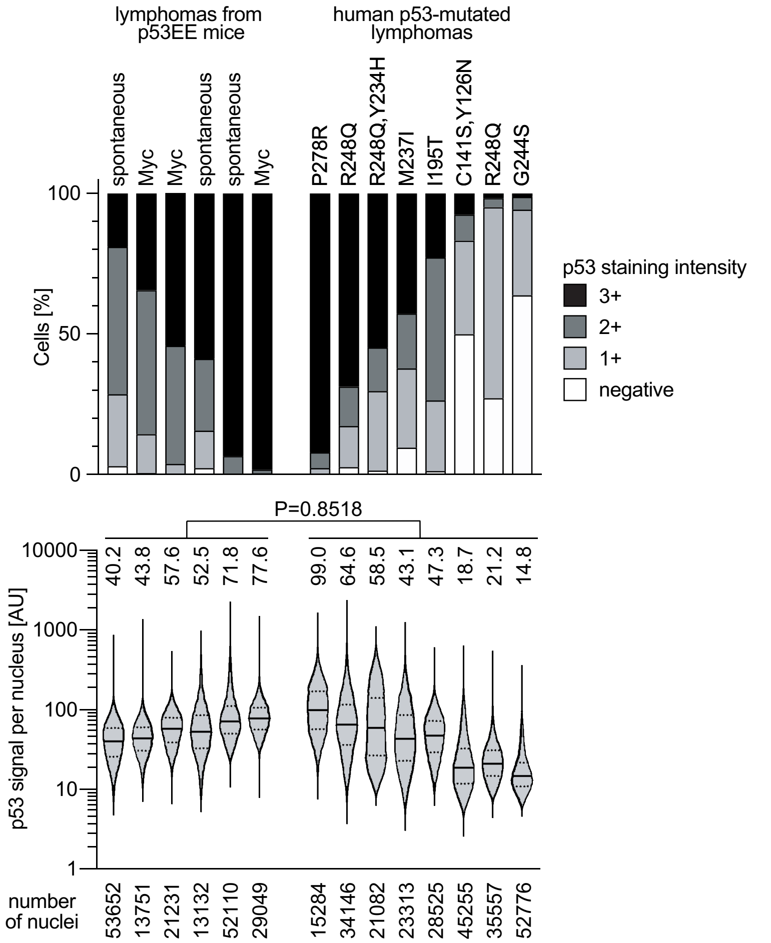

B

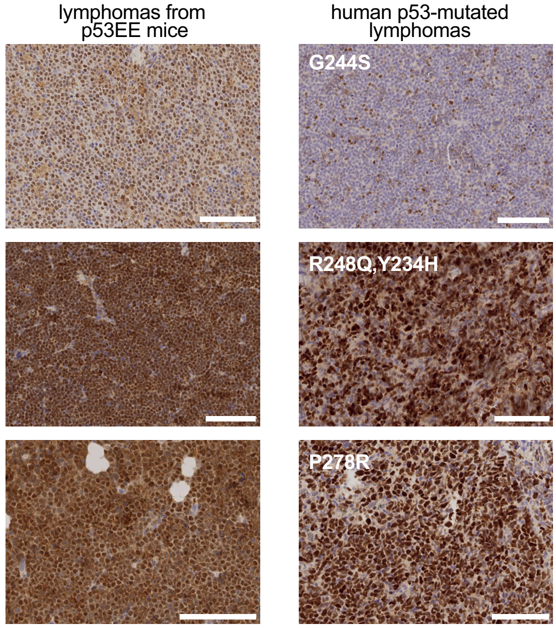

Figure EV4. Mutant p53 expression in p53EE lymphomas compared to human cancer samples.

Spontaneous lymphomas from p53EE mice and human lymphomas with the indicated p53 mutations were stained for p53 using FL-393 antibody (which has comparable affinity for human and murine p53). p53 staining intensity was quantified by automated image analysis.

A Top: Percentage of cells with indicated staining intensity scores for each sample. Bottom: Distribution and mean of cellular staining intensities for each sample. Violin plots were generated with the GraphPad Prism 8 default algorithm (high smoothing) and indicate the median and quartiles with solid and dotted lines, respectively. ns, no significant difference of the mean staining intensity between the two groups of murine and human lymphomas (Mann–Whitney test; unpaired, non-parametric, two-tailed).

B Exemplary images. The analysis demonstrates that p53EE expression in murine lymphomas is within the range for mutant p53 expression in human lymphomas. Scale bar: 100 μm.

 © 2019 The Authors

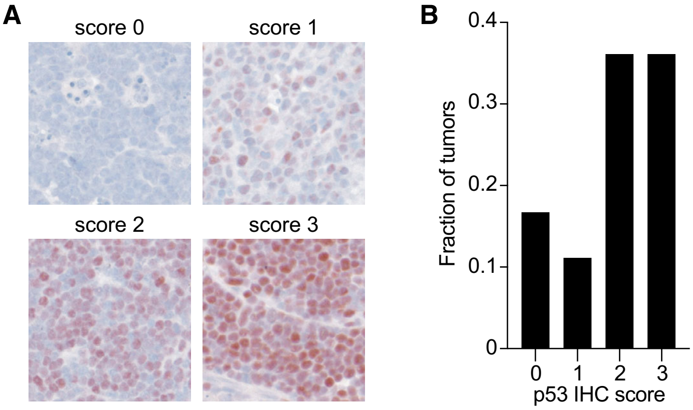

Figure EV5. p53EE expression in tumors from p53$^{EE/EE}$ mice.

A p53 immunohistochemistry images of spontaneous thymic lymphomas from p53$^{EE/EE}$ mice representative of p53 immunostaining scores 0–3.

B Fraction of p53$^{EE/EE}$ mouse tumors with indicated p53 immunostaining scores.

© 2019 The Authors
