# Supplementary material for: Residual apoptotic activity of a tumorigenic p53 mutant improves cancer therapy responses
Source: EMBO J. 2019 Sep 4;38(20):e102096. doi: 10.15252/embj.2019102096 (PMC6792016; doi:10.15252/embj.2019102096)
Supplement: Supplementary file 3 — Table EV1 [file EMBJ-38-e102096-s003.docx]

**Expanded View Table 1. Oligonucleotides**

| Gene/Region | Application | Sense primer | Antisense primer |
| --- | --- | --- | --- |
| Trp53 exon 5-6 | sequencing | tctcttccagtactctcctcc | aattacagacctcgggtggct |
| Trp53 Exon 2 | sequencing | GGACTGCAGGGTCTCAGAAG | TCTCTAGCAGCTGGGCCTAC |
| targeted Trp53-LSL (#A,B) | genotyping | TCTTTGTGAAGGAACCTTACT | CATTCATCAGTTCCATAGGTT |
| targeted Trp53-recombined (#C,D) | genotyping | CCCTGAGAAGAGCAAGGC | AACCAGATCAGGAGGGTCAC |
| Mdm2 | genotyping | CGCCACCAGAAGAGAAACCT | TGTCCCTATGTACCTGTCTCACT |
| Mdm2<tm1.2Mep> | genotyping | GTATTGGGCATGTGTTAGACTGG | CCTGGATTTAATCTGCAGCACTC |
| Eµ-Myc tg | qPCR | GCGGCTCACCTTTAGCATCA | TCCAGCGCATCAGTTCTGTC |
| control locus (Trp53) | qPCR | GCACATGACGGAGGTCGTG | CCAGGCCTAAGAGCAAG |
| mt-Nd4, mouse | qPCR | GCTCCATACCAATCCCCATCA | ACGTAATCTGTTCCGTACGTGT |
| mt-Co1, mouse | qPCR | TCGGAGCCCCAGATATAGCA | TTTCCGGCTAGAGGTGGGTA |
| mt-Cyb, mouse | qPCR | ACCTCAAAGCAACGAAGCCTA | TGGGTGTTCTACTGGTTGGC |
| mt-Nd2, mouse | qPCR | ATCCTCCTGGCCATCGTACT | ATCAGAAGTGGAATGGGGCG |
| p53 RE | EMSA | GGGTAGACATGCCTAGACATGCCTAAGCTCCC | GGGAGCTTAGGCATGTCTAGGCATGTCTACCC |
| Scrambled | EMSA | GGGCCAGCTAGCAGGCAGCATCAGTACTTCCC | GGGAAGTACTGATGCTGCCTGCTAGCTGGCCC |
| **β**-actin, mouse | RTqPCR | CATTGCTGACAGGATGCAGAAGG | TGCTGGAAGGTGGACAGTGAGG |
| Trp53, mouse | RTqPCR | CCTCTGAGCCAGGAGACATT | CAACAGATCGTCCATGCAGT |
| Cdkn1a, mouse | RTqPCR | CAAGAGGCCCAGTACTTCCT | ACACCAGAGTGCAAGACAGC |
| Pmaip1/Noxa, mouse | RTqPCR | GAGTGCACCGGACATAACTG | CTCGTCCTTCAAGTCTGCTG |
| Bbc3/Puma, mouse | RTqPCR | GTACGAGCGGCGGAGACAAG | GCACCTAGTTGGGCTCCATTTCTG |
| Bax, mouse | RTqPCR | TAGCAAACTGGTGCTCAAGG | TCTTGGATCCAGACAAGCAG |
| Cdkn2a/Arf, mouse | RTqPCR | TGGTCACTGTGAGGATTCAGC | GTTGCCCATCATCATCACCTGG |
| Cdkn2a/p16, mouse | RTqPCR | GCCCAACGCCCCGAACTCTTTC | GCGACGTTCCCAGCGGTACACA |
| Ccng1, mouse | RTqPCR | AAGTGCTCCAAACCTAACGG | GAATCGTTGGGAGGTGAGTT |
| Mdm2, mouse | RTqPCR | CTAGCTTCTCCCTGAATGCC | TTGCACACGTGAAACATGAC |
| Sfn, mouse | RTqPCR | CCTGCTTTCCGTAGCTTACA | TCCCGGTACTCTTTCACCTC |
| Hmox1, mouse | RTqPCR | AAGCCGAGAATGCTGAGTTCA | GCCGTGTAGATATGGTACAAGGA |
| Nqo1, mouse | RTqPCR | AGGATGGGAGGTACTCGAATC | AGGCGTCCTTCCTTATATGCTA |
| Gpx1, mouse | RTqPCR | CGGGGTGGTGCTCGGTTTCCCG | CCAGGTCGGACGTACTTGAGG |
| Slc7a11, mouse | RTqPCR | GCCATGGTCAGAAAGCCAGT | GCATAGGACAGGGCTCCAAAA |
| Sod3, mouse | RTqPCR | CCTTCTTGTTCTACGGCTTGC | TCGCCTATCTTCTCAACCAGG |
| CDKN1A, human | RTqPCR | TGGAGACTCTCAGGGTCGAAA | CCGGCGTTTGGAGTGGTA |
| BBC3/Puma, human | RTqPCR | ACCTCAACGCACAGTACGAG | GAGATTGTACAGGACCCTCCA |
| BAX, human | RTqPCR | GGGTTGTCGCCCTTTTCTACTT | AGCCCATGATGGTTCTGATCAG |
| sgRNA1 Trp53 | CRISPR-Cas9 | CACCGACCCTGTCACCGAGACCCC | AAACGGGGTCTCGGTGACAGGGTC |
| sgRNA2 Trp53 | CRISPR-Cas9 | CACCGCAGGAGCTCCTGACACTCGG | AAACCCGAGTGTCAGGAGCTCCTGC |
| sgRNA GFP (control) | CRISPR-Cas9 | CACCGGGGCGAGGAGCTGTTCACCG | AAACCGGTGAACAGCTCCTCGCCCC |
