# Supplementary figures and images for: Residual apoptotic activity of a tumorigenic p53 mutant improves cancer therapy responses
Source: EMBO J. 2019 Sep 4;38(20):e102096. doi: 10.15252/embj.2019102096 (PMC6792016; doi:10.15252/embj.2019102096)

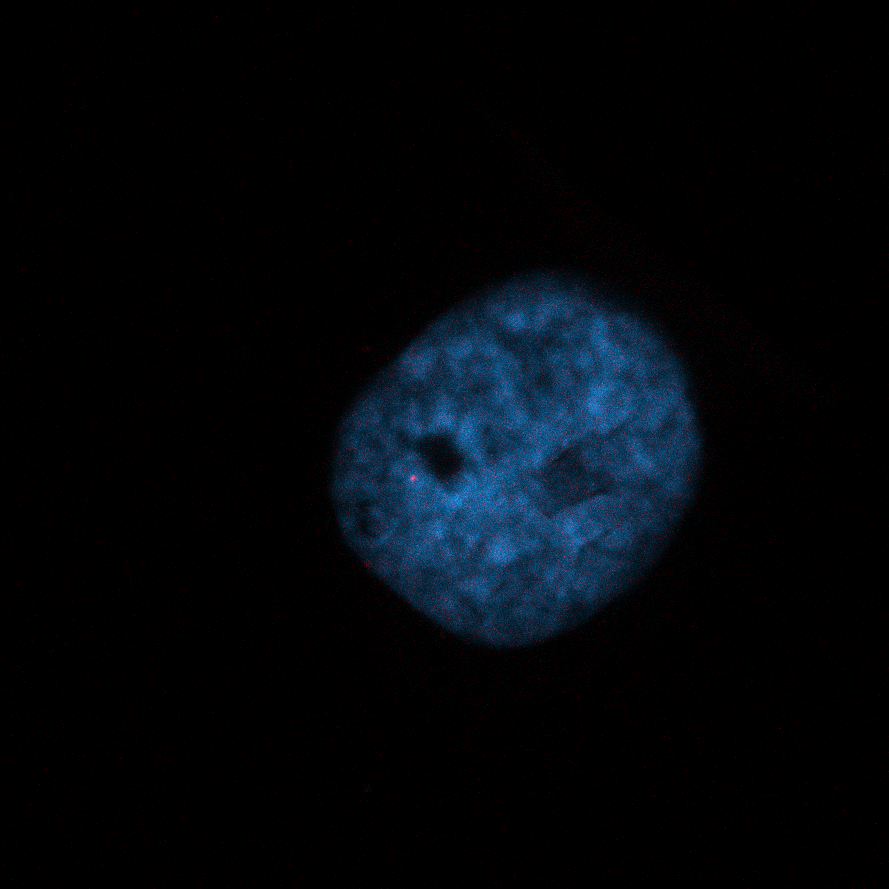

Supplement: Supplementary file 5 — Source Data for Figure 5E [file EMBJ-38-e102096-s004.zip › Soure_Data_Figure5E/Figure_5E_Source_Data/95549_2_additional_figure_data_1533114_pv7mr2.tiff]

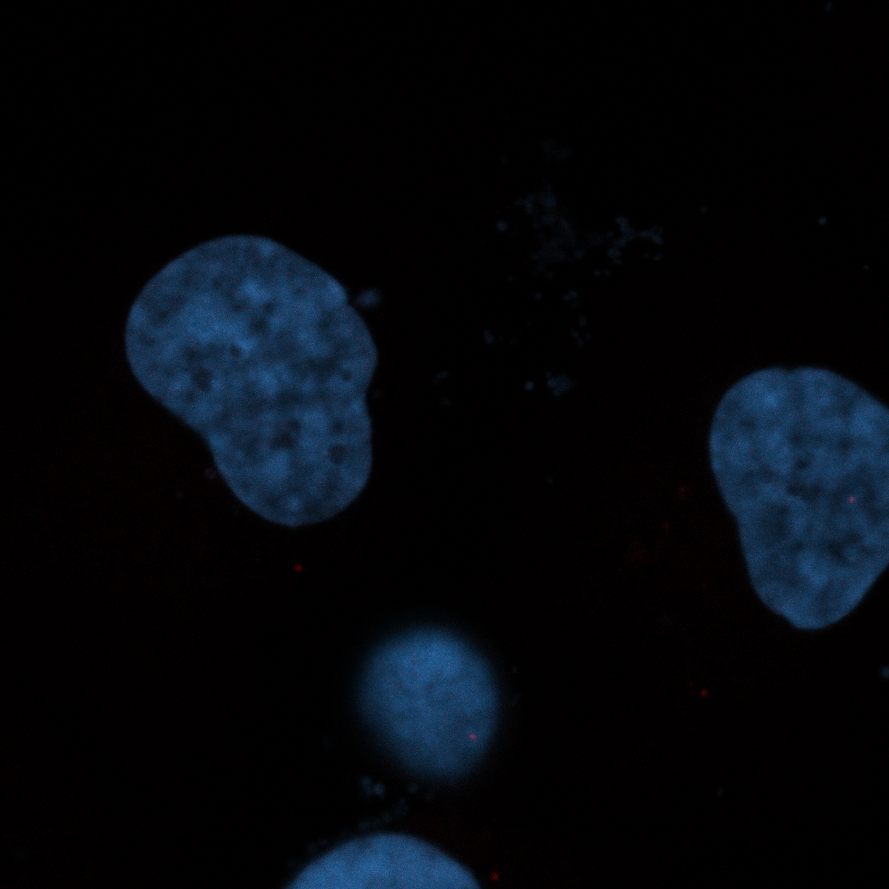

Supplement: Supplementary file 5 — Source Data for Figure 5E [file EMBJ-38-e102096-s004.zip › Soure_Data_Figure5E/Figure_5E_Source_Data/95549_2_additional_figure_data_1533115_pv7mr2.tiff]

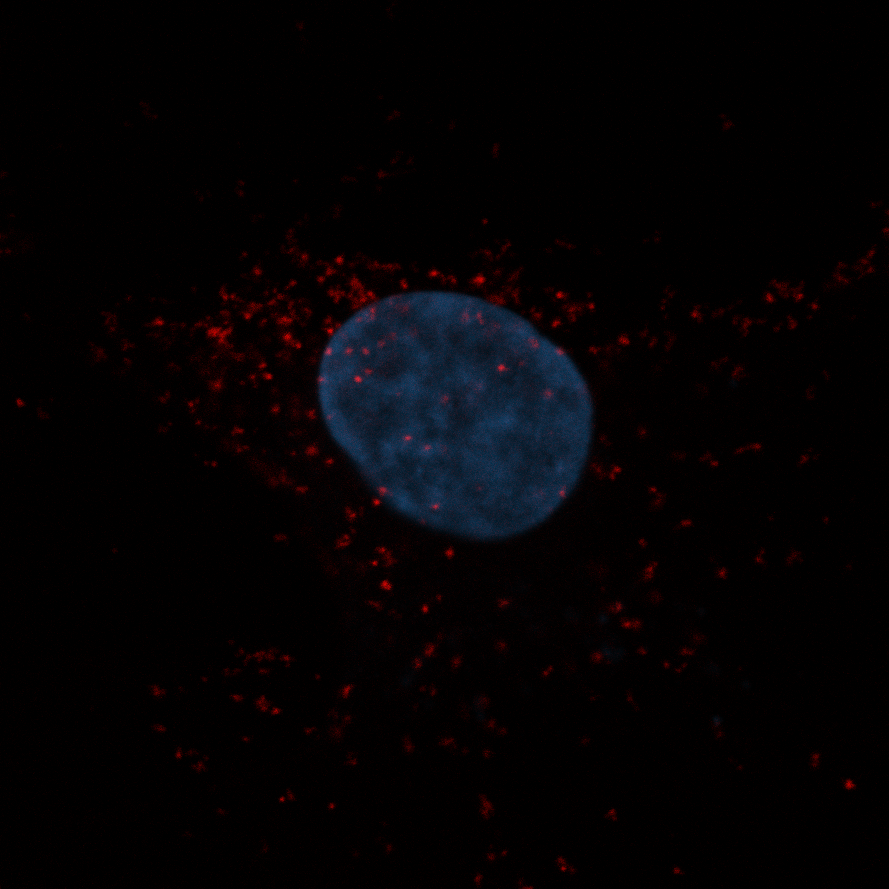

Supplement: Supplementary file 5 — Source Data for Figure 5E [file EMBJ-38-e102096-s004.zip › Soure_Data_Figure5E/Figure_5E_Source_Data/95549_2_additional_figure_data_1533116_pv7mr2.tiff]

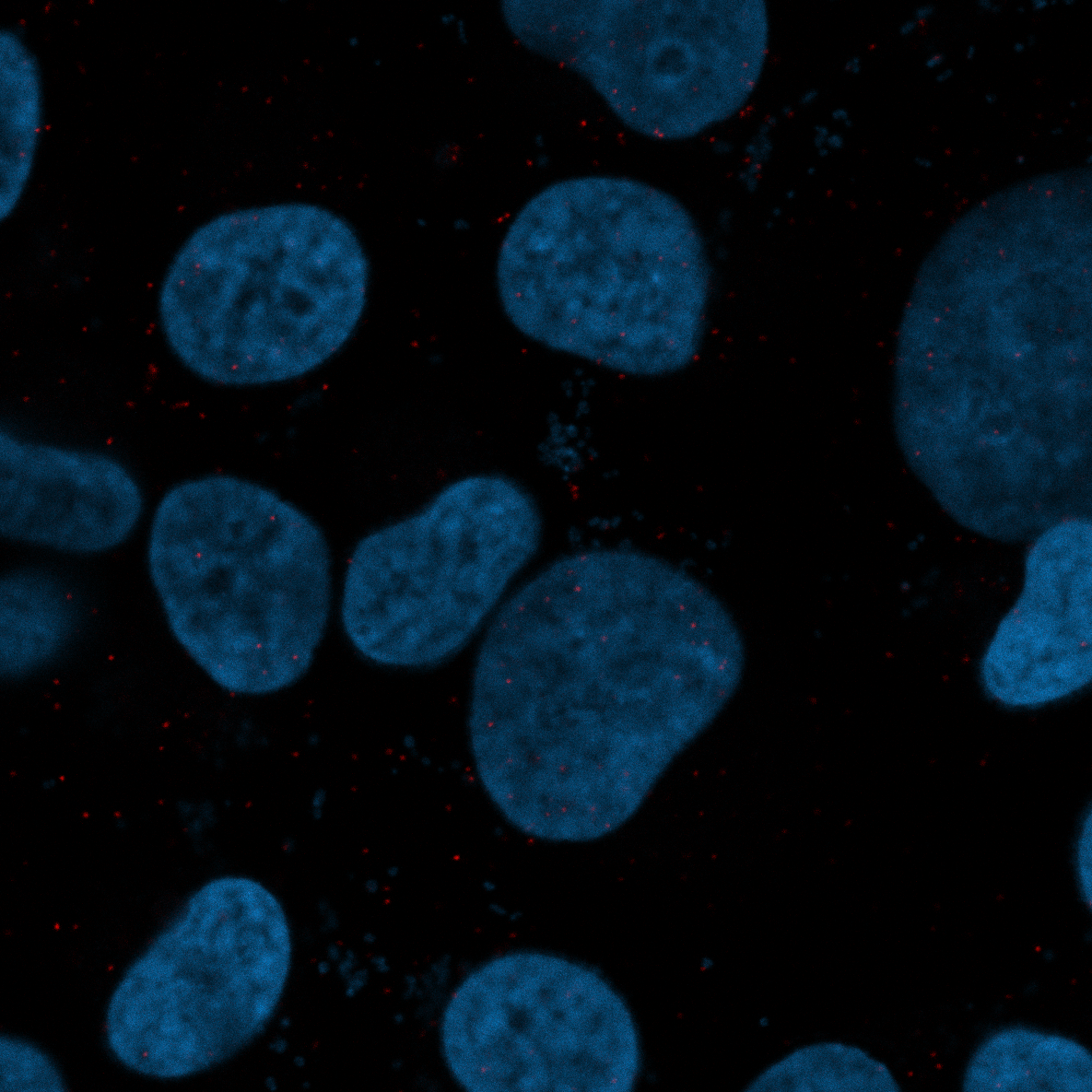

Supplement: Supplementary file 5 — Source Data for Figure 5E [file EMBJ-38-e102096-s004.zip › Soure_Data_Figure5E/Figure_5E_Source_Data/95549_2_additional_figure_data_1533117_pv7mr3.tiff]
