# Supplementary figures and images for: Author Correction: Residual apoptotic activity of a tumorigenic p53 mutant improves cancer therapy responses
Source: EMBO J. 2026 Mar 24;45(9):3332–5. doi: 10.1038/s44318-026-00758-4 (PMC13144674; doi:10.1038/s44318-026-00758-4)

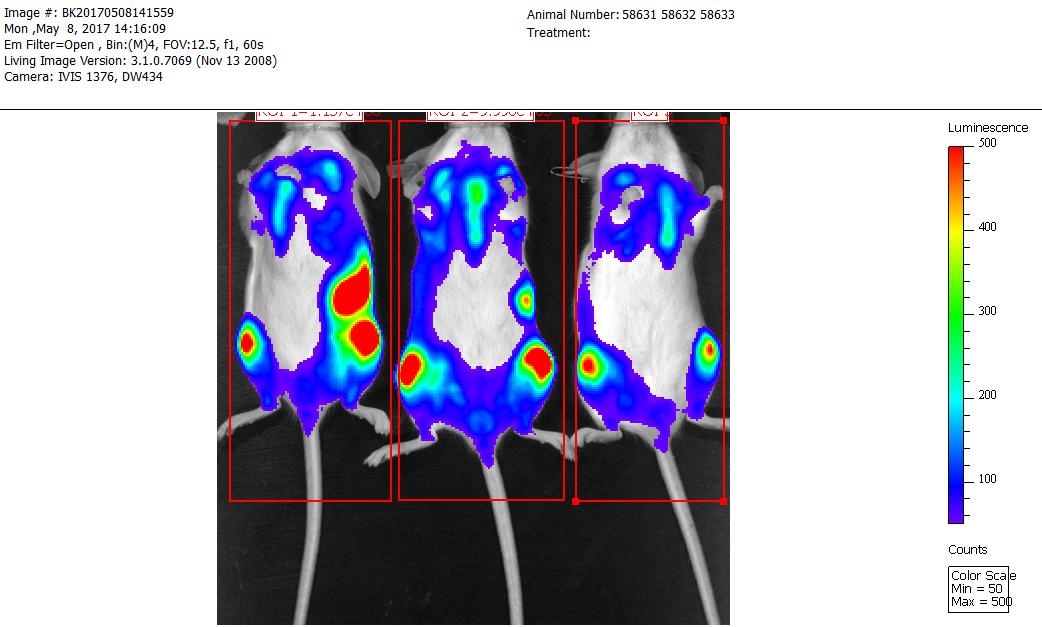

Supplement: Supplementary file 1 — Figure 7G Source Data [file 44318_2026_758_MOESM1_ESM.zip › EE/EE untreated 1,2, therapy 1 d0.jpg]

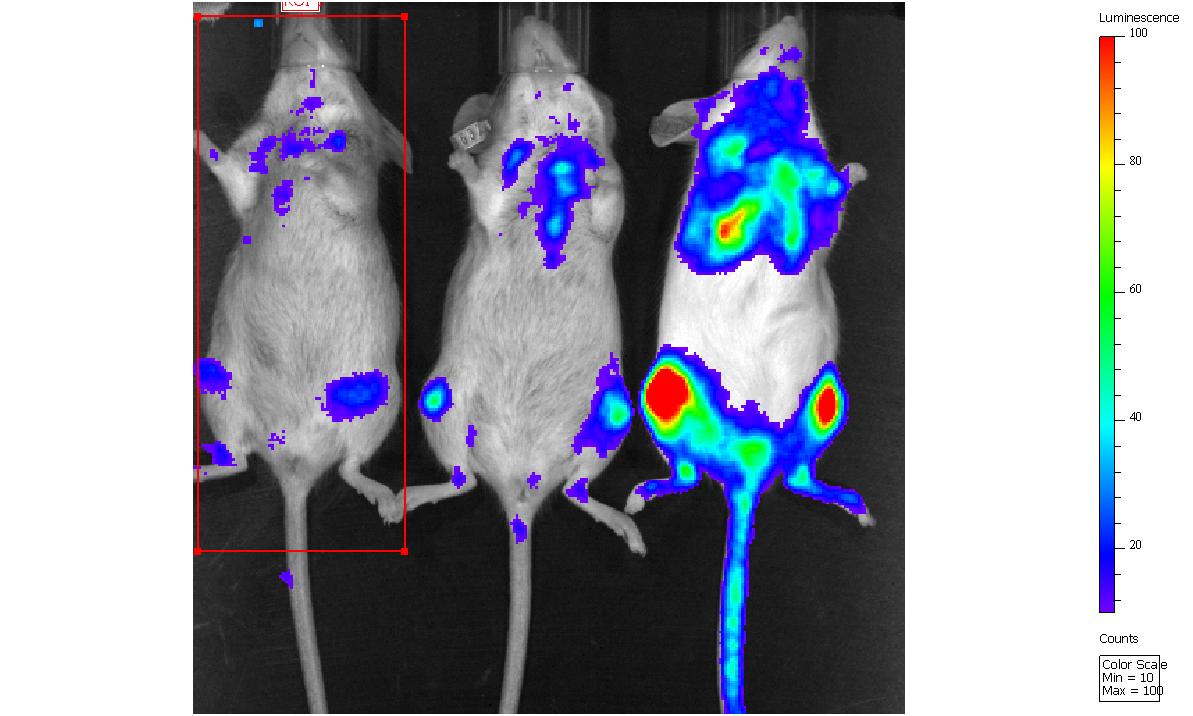

Supplement: Supplementary file 1 — Figure 7G Source Data [file 44318_2026_758_MOESM1_ESM.zip › EE/EE therapy 2 d7.jpg]

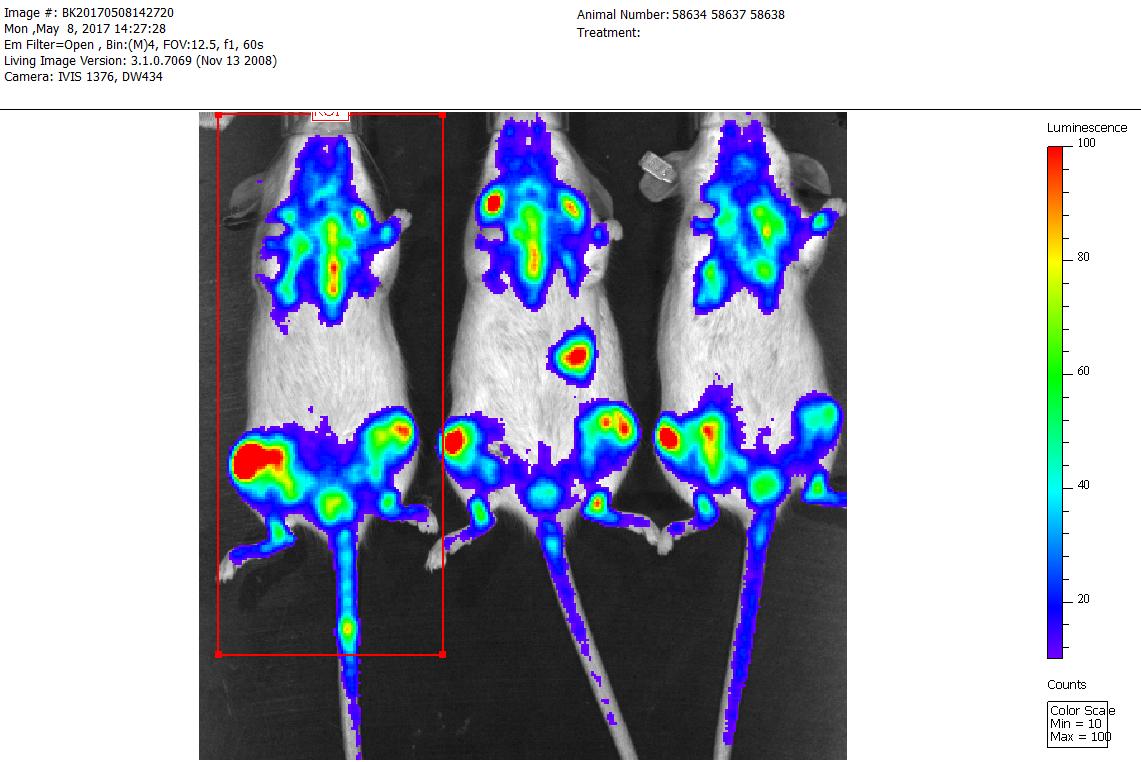

Supplement: Supplementary file 1 — Figure 7G Source Data [file 44318_2026_758_MOESM1_ESM.zip › EE/EE therapy 2 d0.jpg]

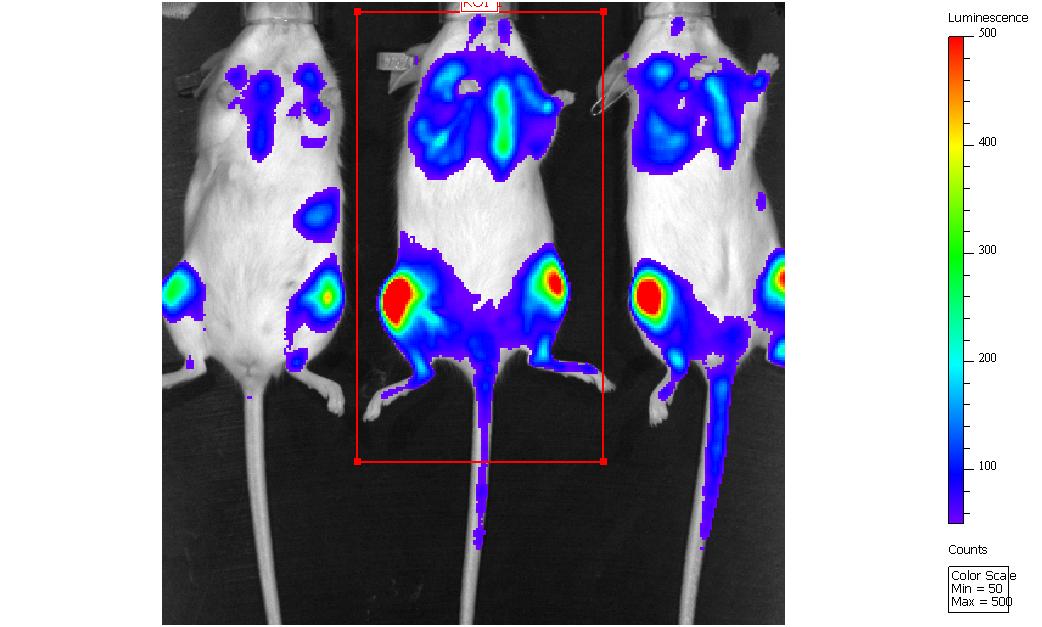

Supplement: Supplementary file 1 — Figure 7G Source Data [file 44318_2026_758_MOESM1_ESM.zip › EE/EE therapy 4 d0.jpg]

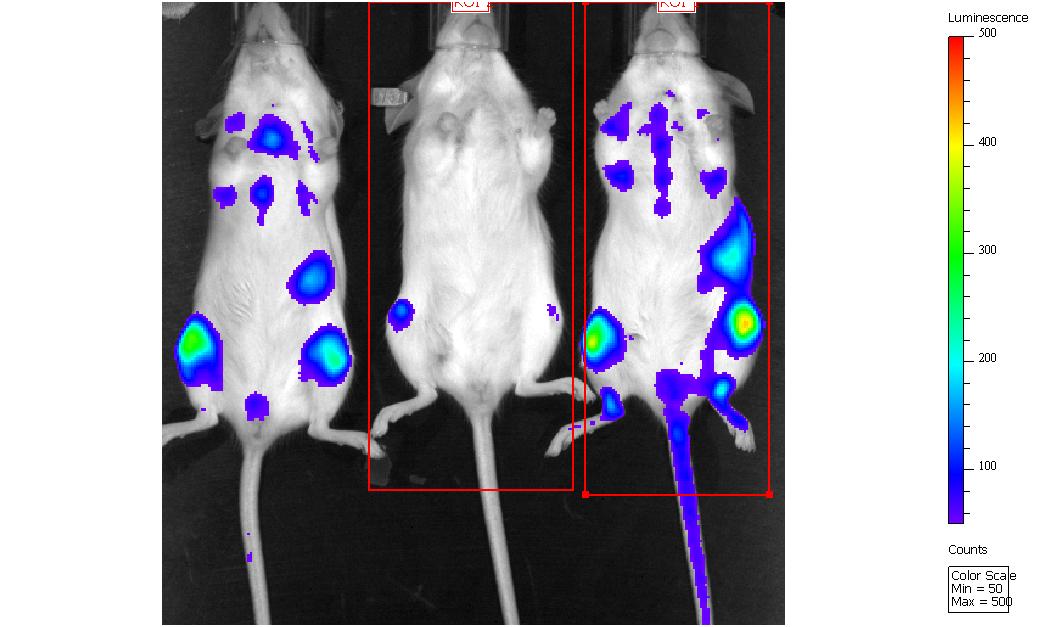

Supplement: Supplementary file 1 — Figure 7G Source Data [file 44318_2026_758_MOESM1_ESM.zip › EE/EE therapy 3,4 d14.jpg]

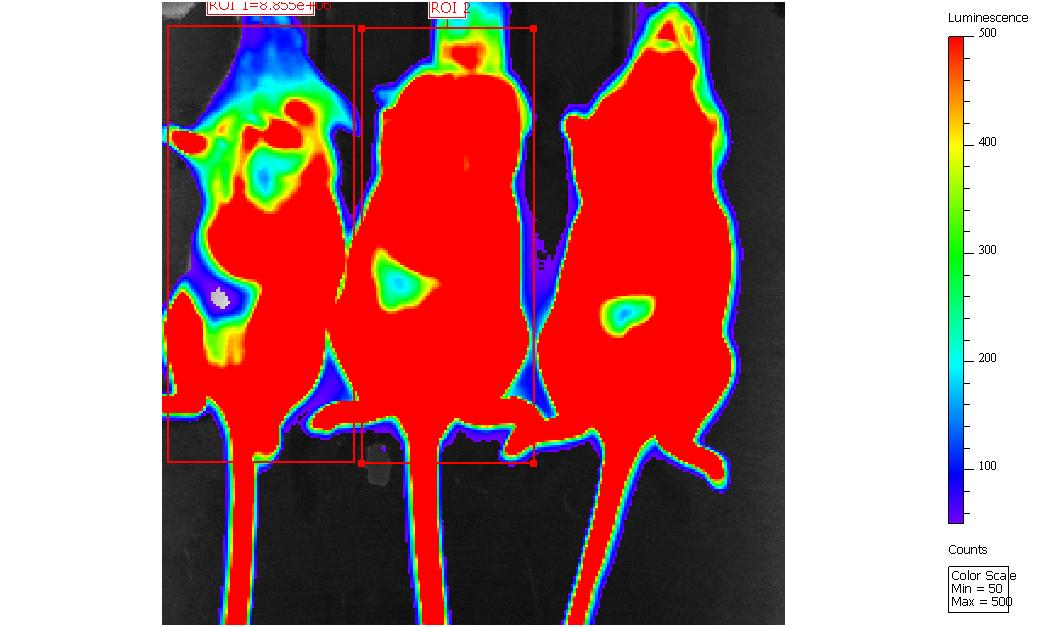

Supplement: Supplementary file 1 — Figure 7G Source Data [file 44318_2026_758_MOESM1_ESM.zip › EE/EE untreated 1,2 d14.jpg]

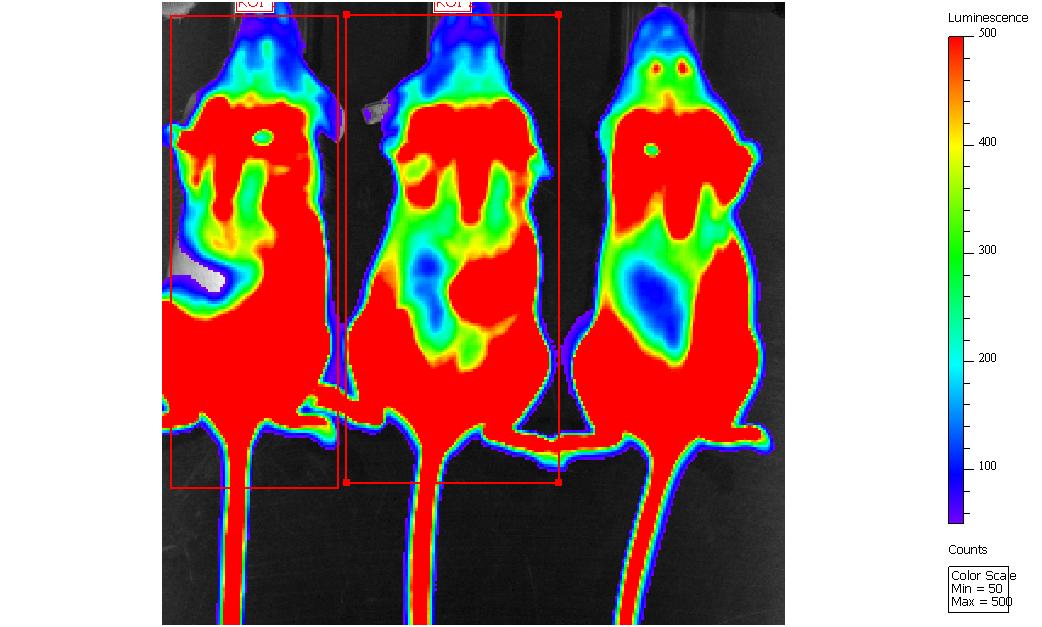

Supplement: Supplementary file 1 — Figure 7G Source Data [file 44318_2026_758_MOESM1_ESM.zip › EE/EE untreated 1,2 d7.jpg]

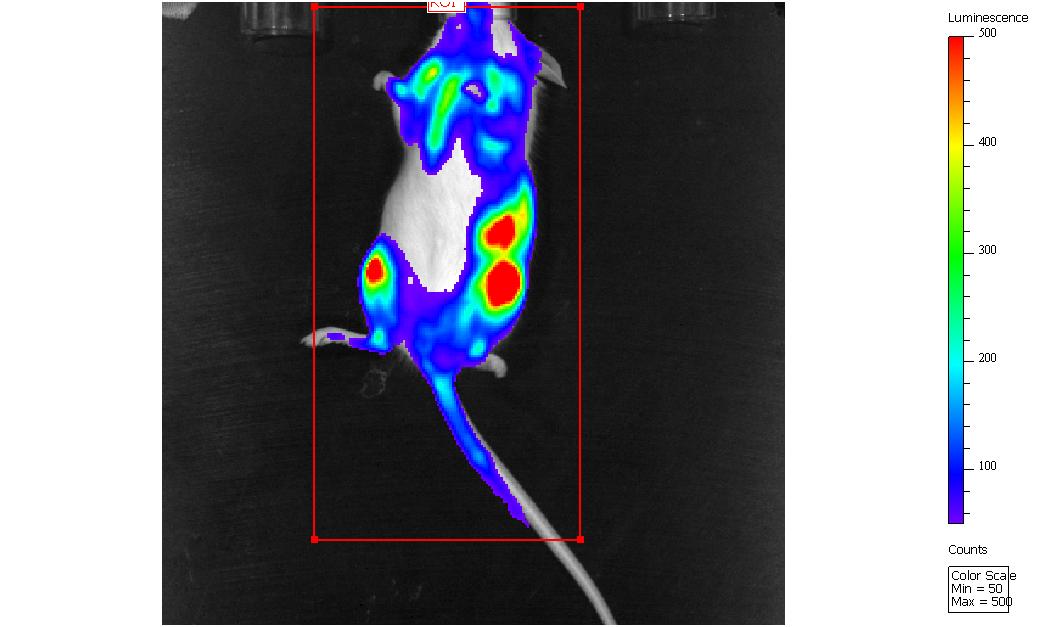

Supplement: Supplementary file 1 — Figure 7G Source Data [file 44318_2026_758_MOESM1_ESM.zip › EE/EE therapy 3 d0.jpg]

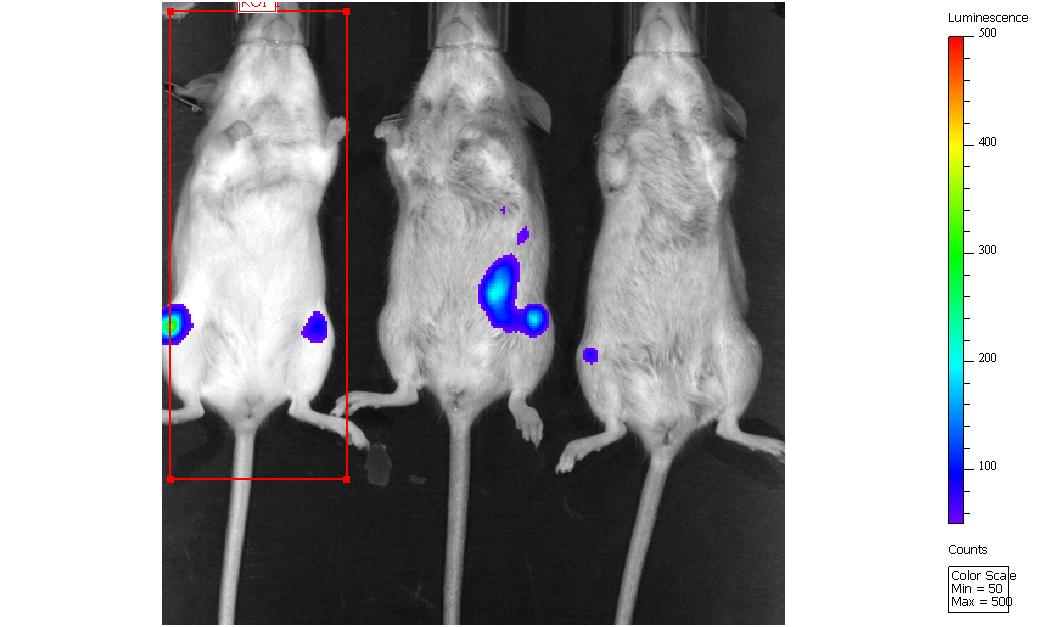

Supplement: Supplementary file 1 — Figure 7G Source Data [file 44318_2026_758_MOESM1_ESM.zip › EE/EE therapy 1 d14.jpg]

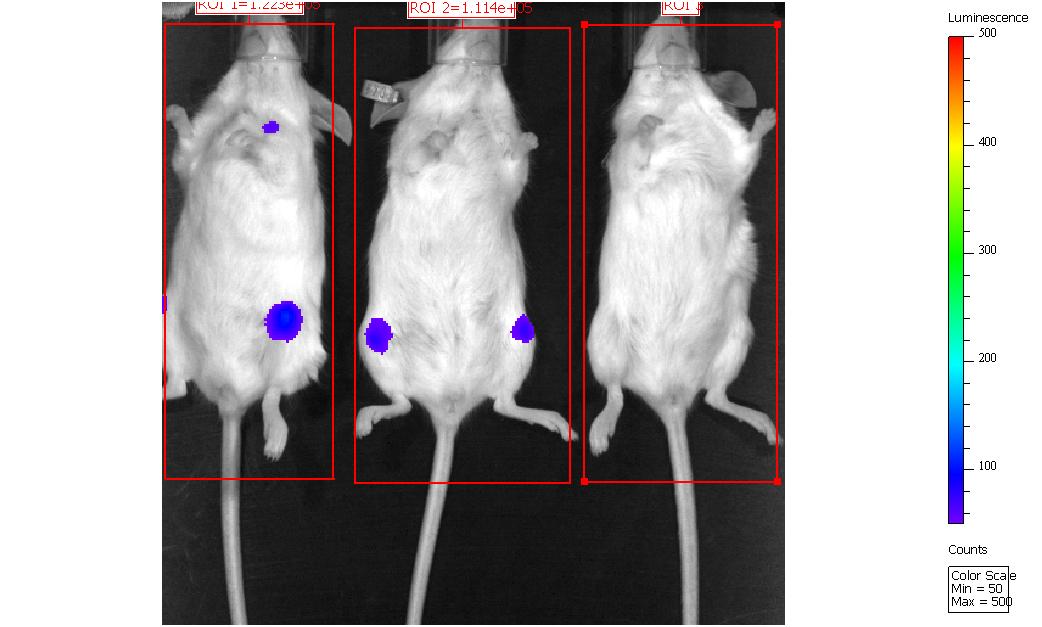

Supplement: Supplementary file 1 — Figure 7G Source Data [file 44318_2026_758_MOESM1_ESM.zip › EE/EE therapy 1, 4, 3 d7.jpg]

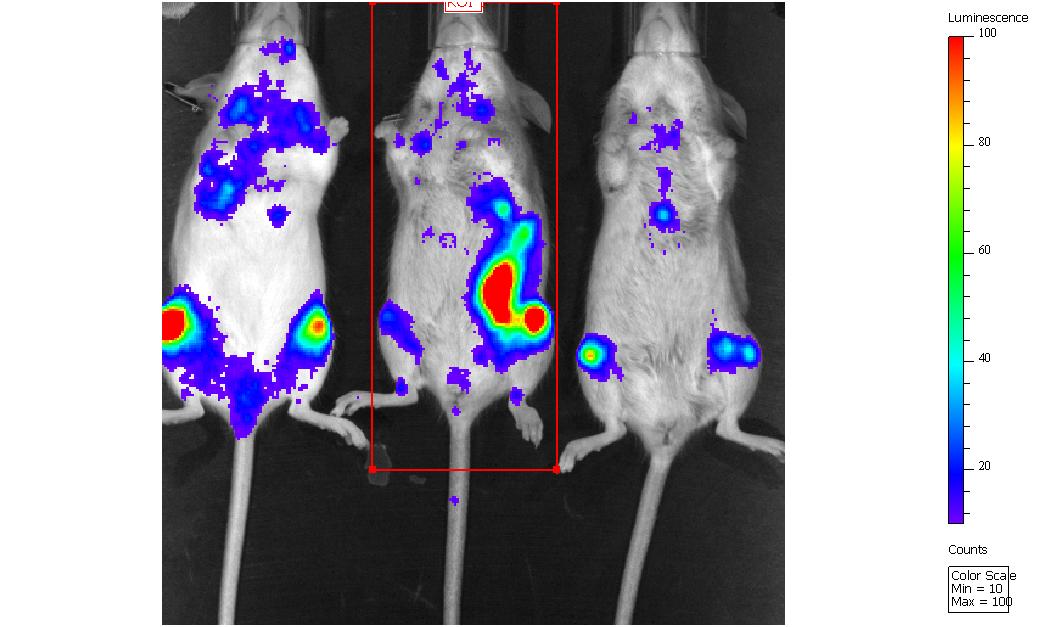

Supplement: Supplementary file 1 — Figure 7G Source Data [file 44318_2026_758_MOESM1_ESM.zip › EE/EE therapy 2 d14.jpg]

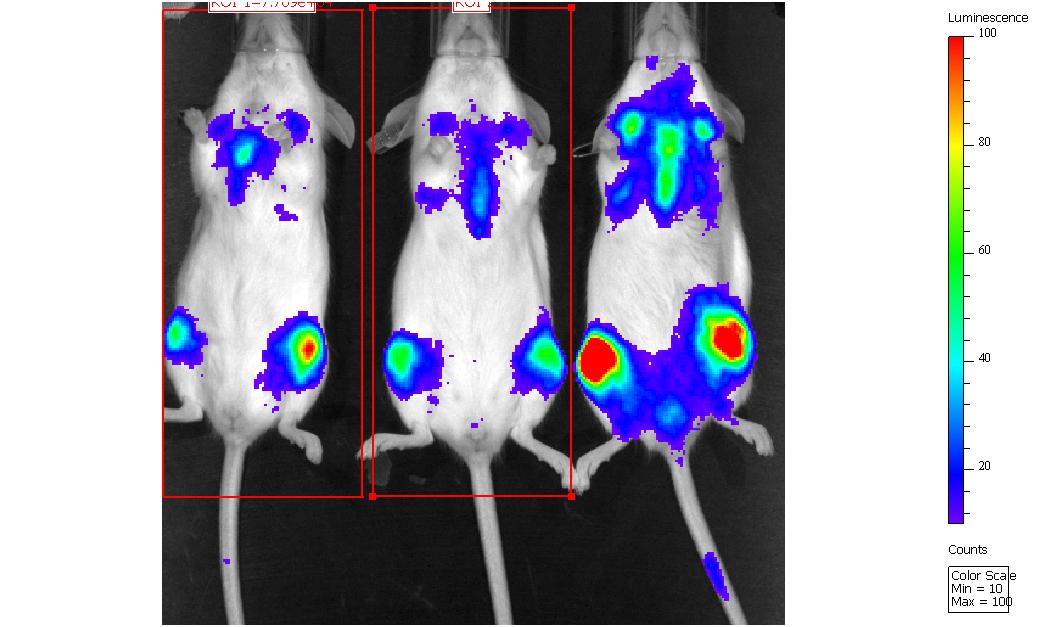

Supplement: Supplementary file 1 — Figure 7G Source Data [file 44318_2026_758_MOESM1_ESM.zip › p53 ko/LSL untreated 1, therapy 1 d0.jpg]

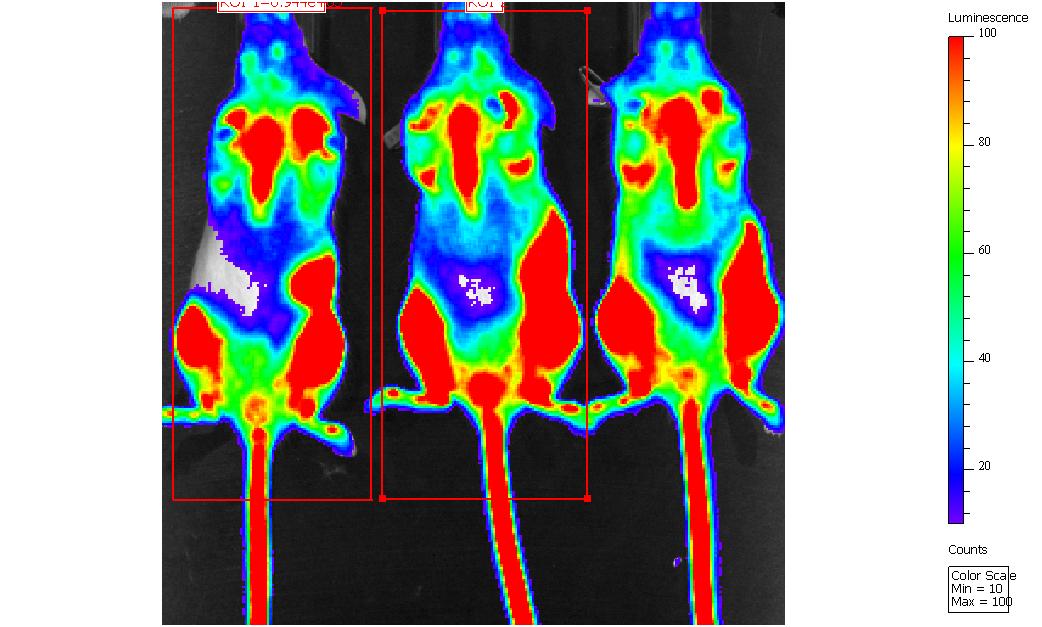

Supplement: Supplementary file 1 — Figure 7G Source Data [file 44318_2026_758_MOESM1_ESM.zip › p53 ko/LSL untreated 1, therapy 1 d7.jpg]

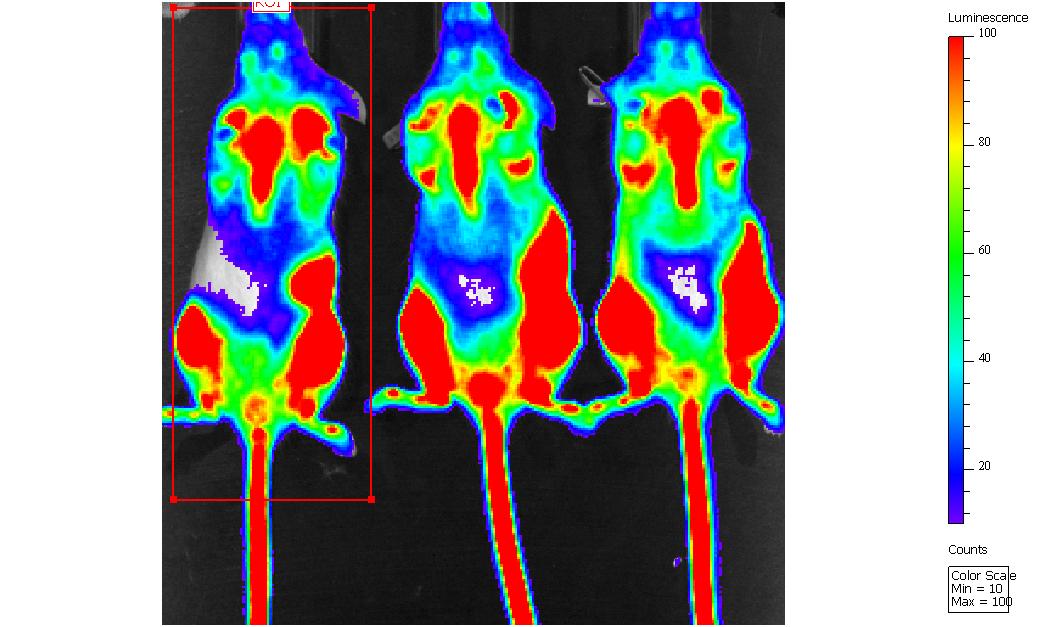

Supplement: Supplementary file 1 — Figure 7G Source Data [file 44318_2026_758_MOESM1_ESM.zip › p53 ko/LSL untreated 1 d7.jpg]

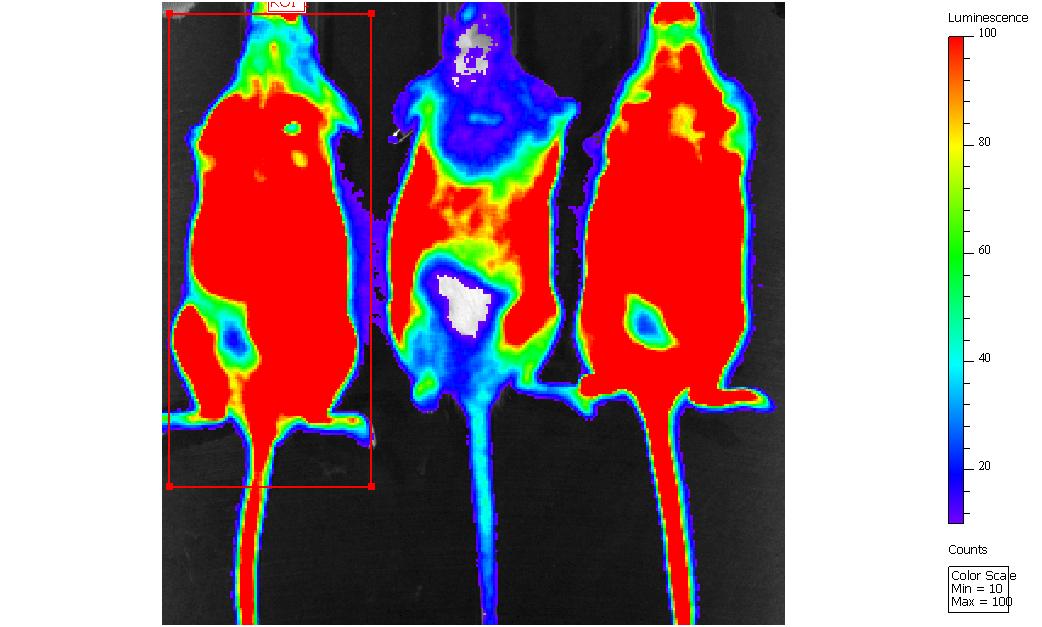

Supplement: Supplementary file 1 — Figure 7G Source Data [file 44318_2026_758_MOESM1_ESM.zip › p53 ko/LSL therapy 3 d14.jpg]

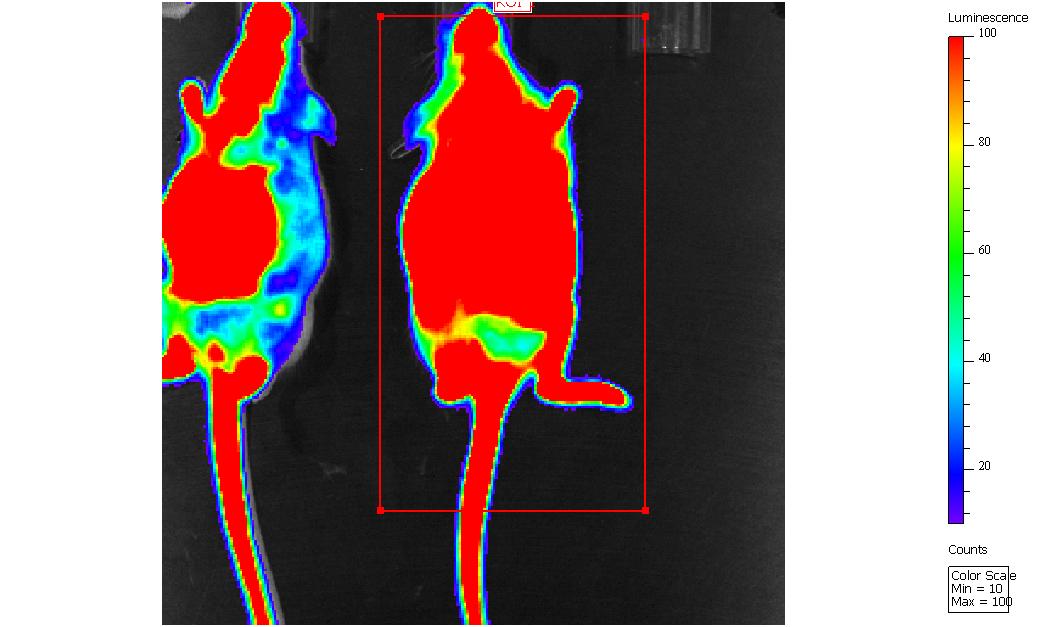

Supplement: Supplementary file 1 — Figure 7G Source Data [file 44318_2026_758_MOESM1_ESM.zip › p53 ko/LSL untreated 2 d14.jpg]

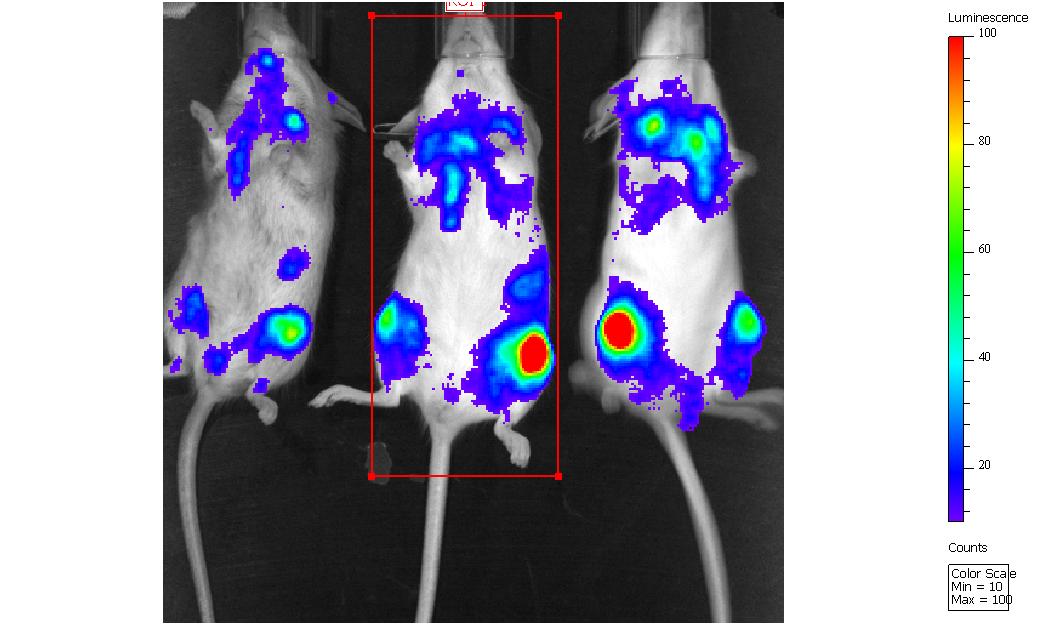

Supplement: Supplementary file 1 — Figure 7G Source Data [file 44318_2026_758_MOESM1_ESM.zip › p53 ko/LSL therapy 4 d0.jpg]

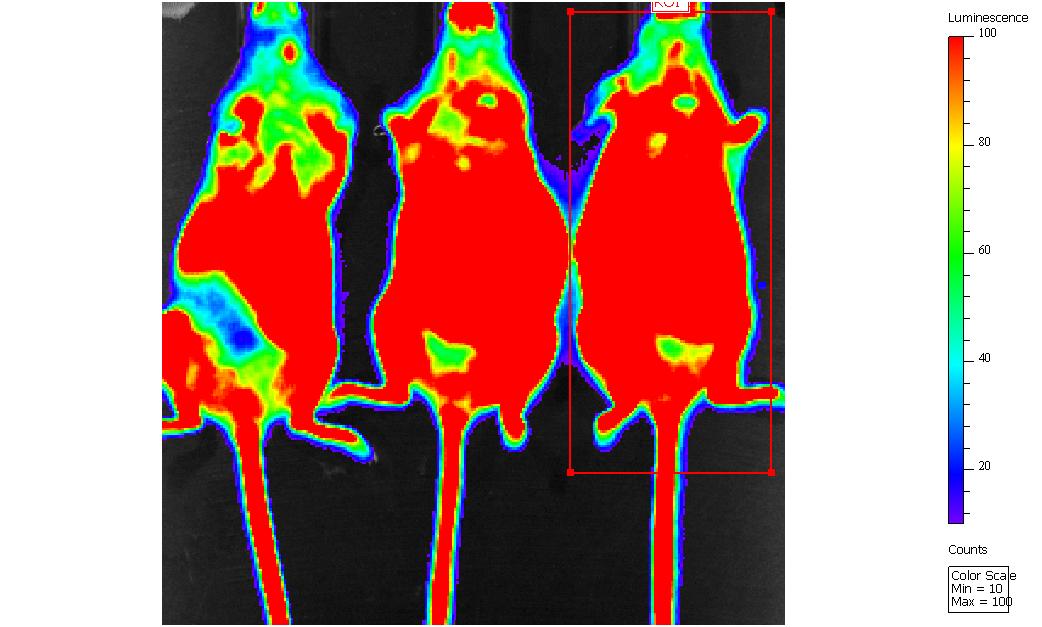

Supplement: Supplementary file 1 — Figure 7G Source Data [file 44318_2026_758_MOESM1_ESM.zip › p53 ko/LSL therapy 2 d14.jpg]

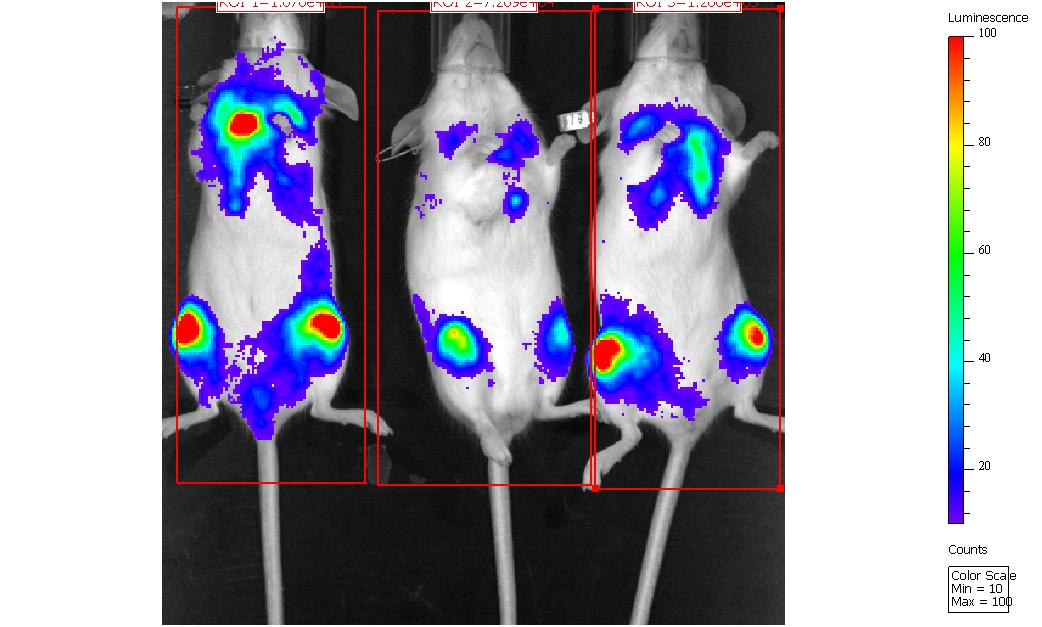

Supplement: Supplementary file 1 — Figure 7G Source Data [file 44318_2026_758_MOESM1_ESM.zip › p53 ko/LSL therapy 2,3, untreated 2 d0.jpg]

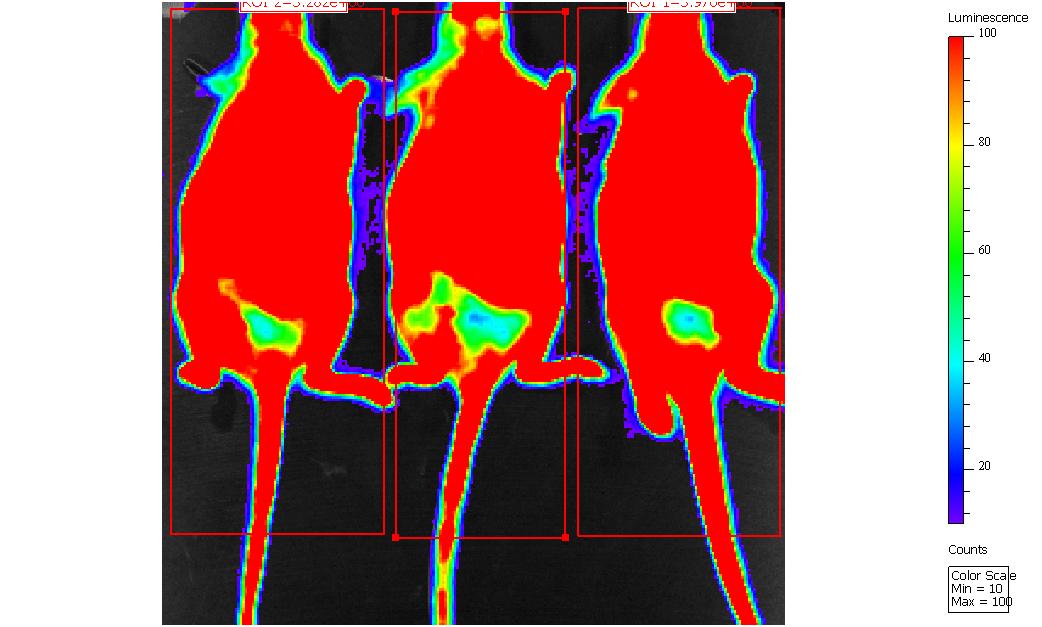

Supplement: Supplementary file 1 — Figure 7G Source Data [file 44318_2026_758_MOESM1_ESM.zip › p53 ko/LSL therapy 1, 4 untreated 1 d14.jpg]

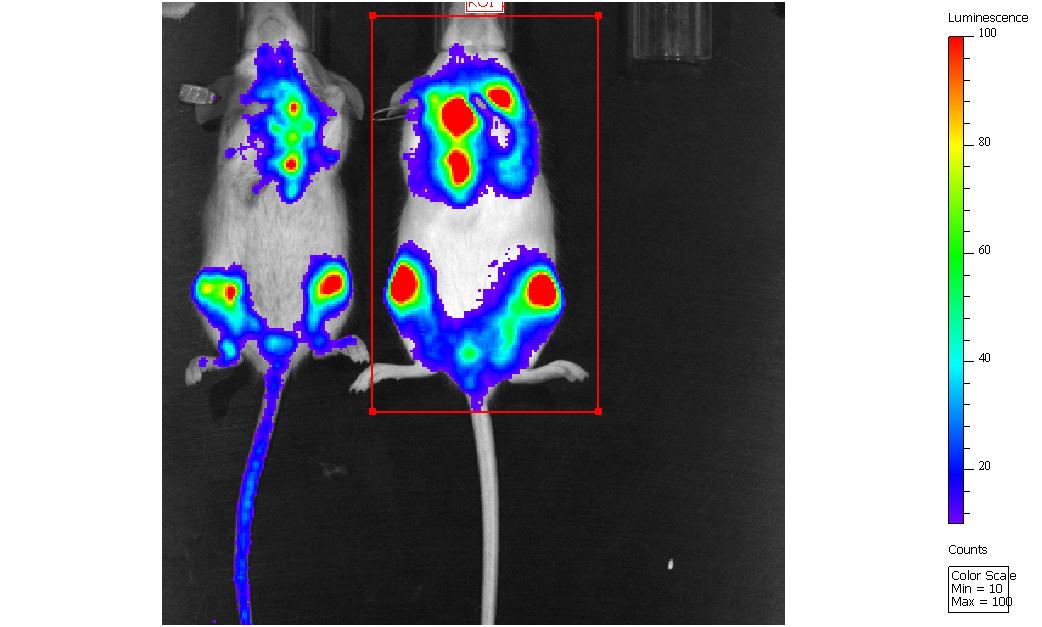

Supplement: Supplementary file 1 — Figure 7G Source Data [file 44318_2026_758_MOESM1_ESM.zip › p53 ko/LSL therapy 3 d7.jpg]

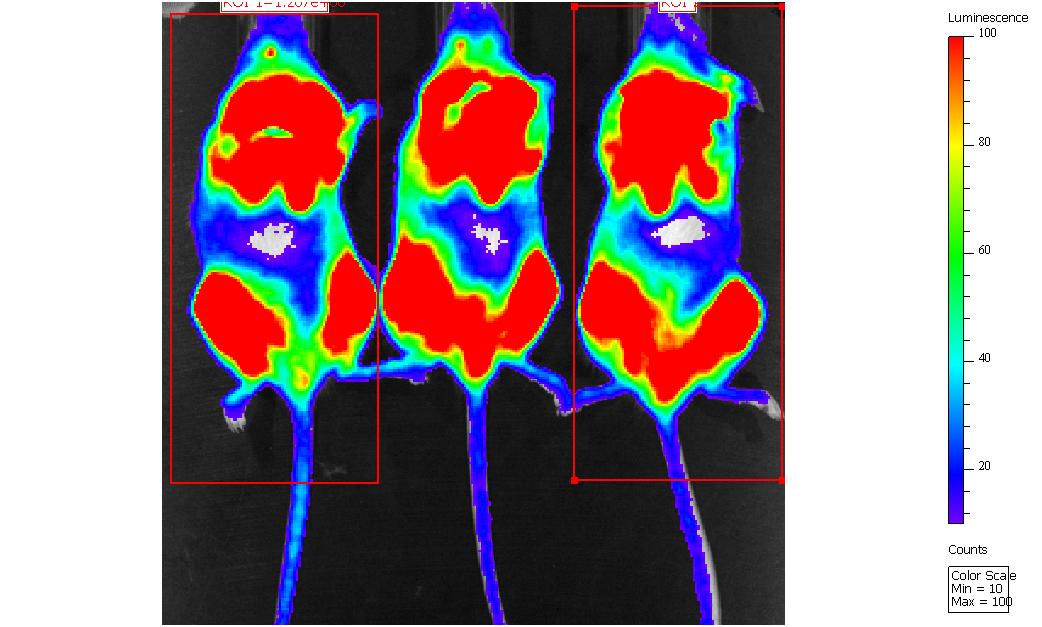

Supplement: Supplementary file 1 — Figure 7G Source Data [file 44318_2026_758_MOESM1_ESM.zip › p53 ko/LSL therapy 4,2 d7.jpg]

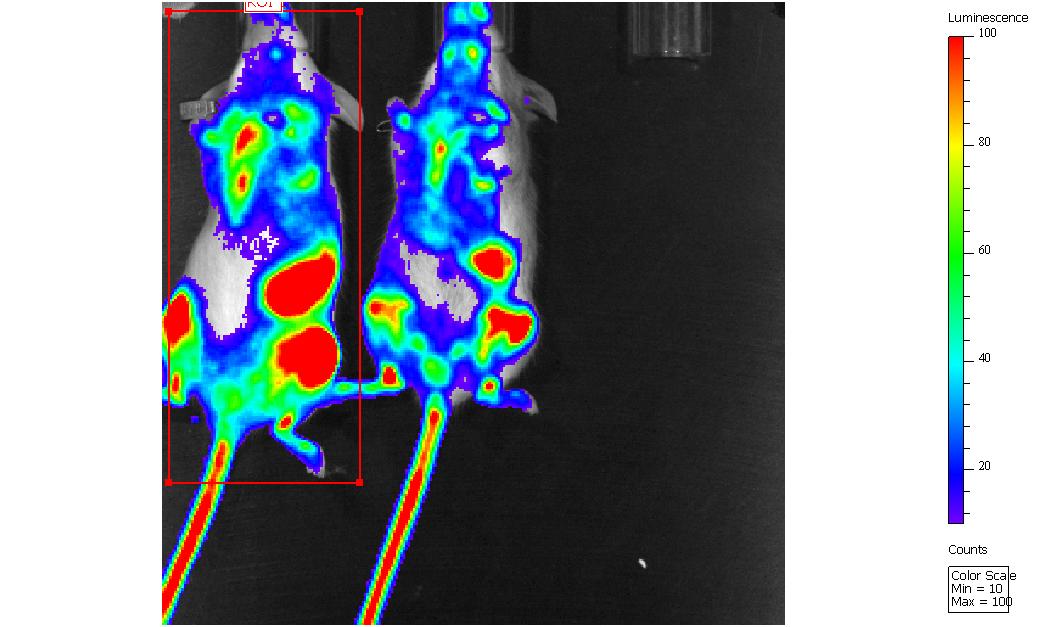

Supplement: Supplementary file 1 — Figure 7G Source Data [file 44318_2026_758_MOESM1_ESM.zip › p53 ko/LSL untreated 2 d7.jpg]

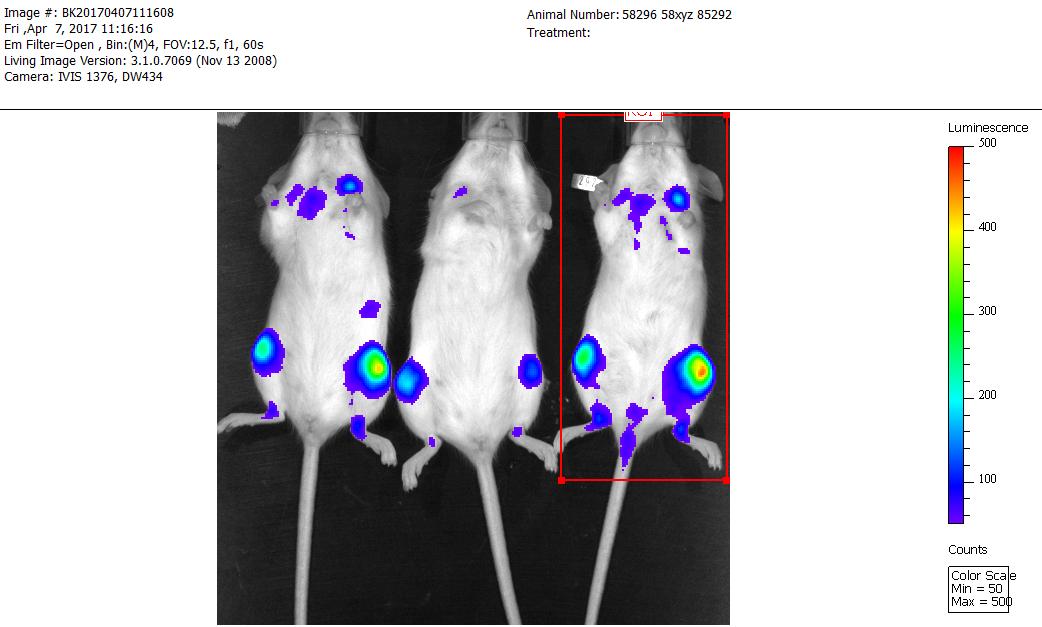

Supplement: Supplementary file 1 — Figure 7G Source Data [file 44318_2026_758_MOESM1_ESM.zip › wt/wt therapy 1 d0.jpg]

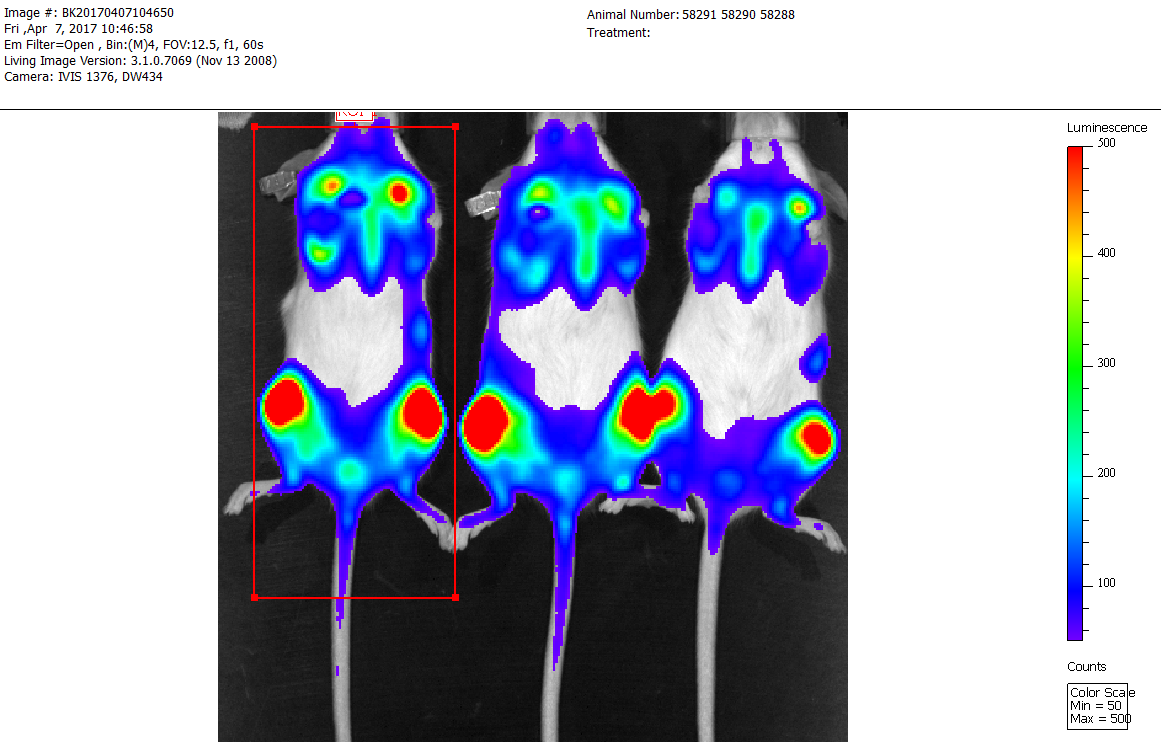

Supplement: Supplementary file 1 — Figure 7G Source Data [file 44318_2026_758_MOESM1_ESM.zip › wt/wt untr 2 d0.PNG]

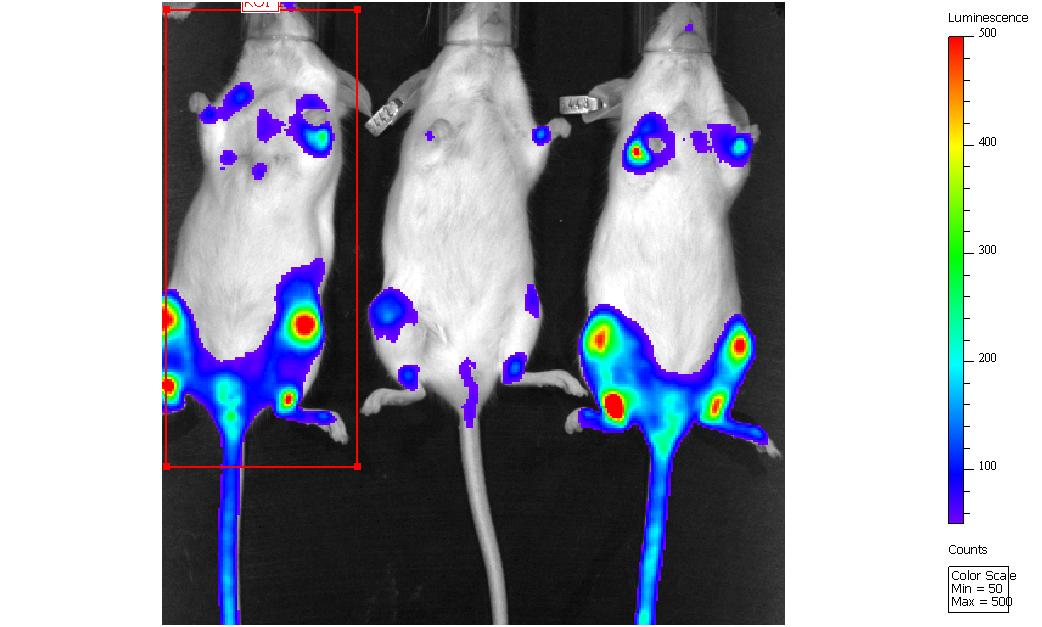

Supplement: Supplementary file 1 — Figure 7G Source Data [file 44318_2026_758_MOESM1_ESM.zip › wt/wt therapy 4 d14.jpg]

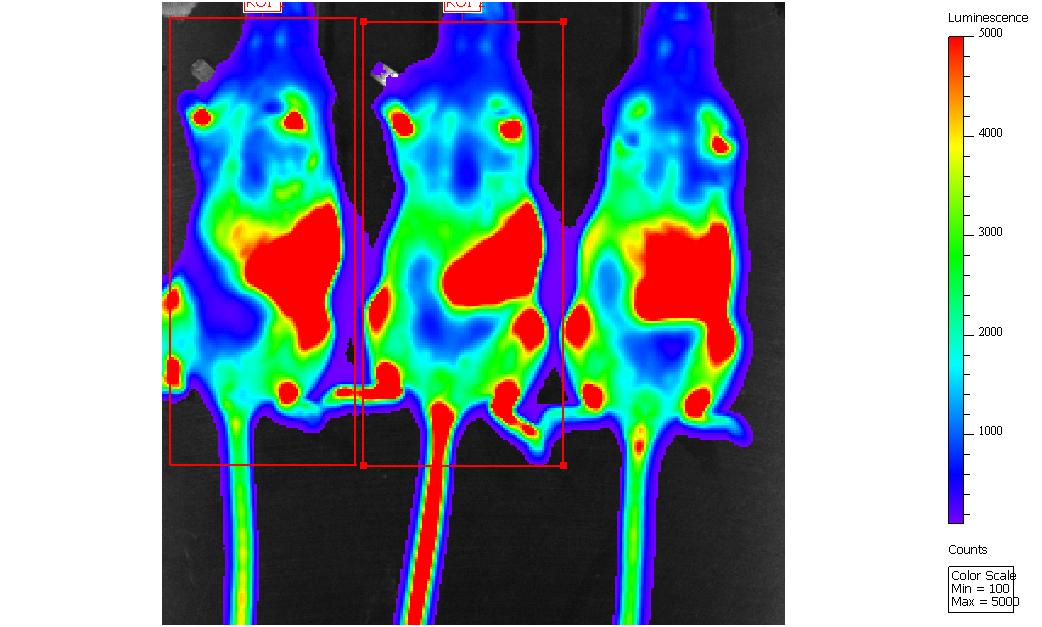

Supplement: Supplementary file 1 — Figure 7G Source Data [file 44318_2026_758_MOESM1_ESM.zip › wt/wt untr 1 and 2 d14.jpg]

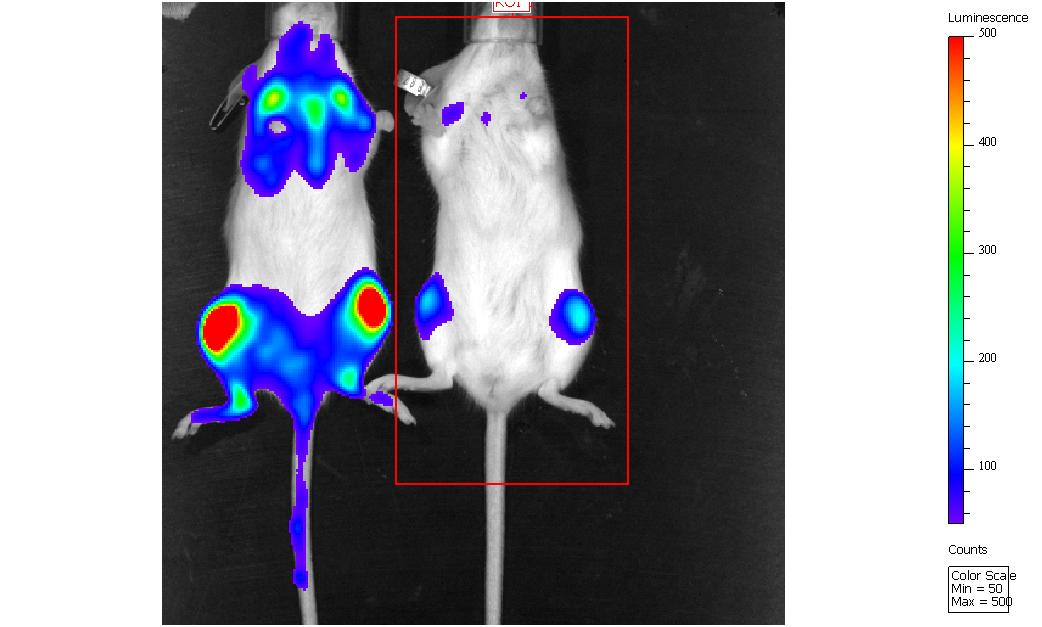

Supplement: Supplementary file 1 — Figure 7G Source Data [file 44318_2026_758_MOESM1_ESM.zip › wt/wt untr 1 d0.jpg]

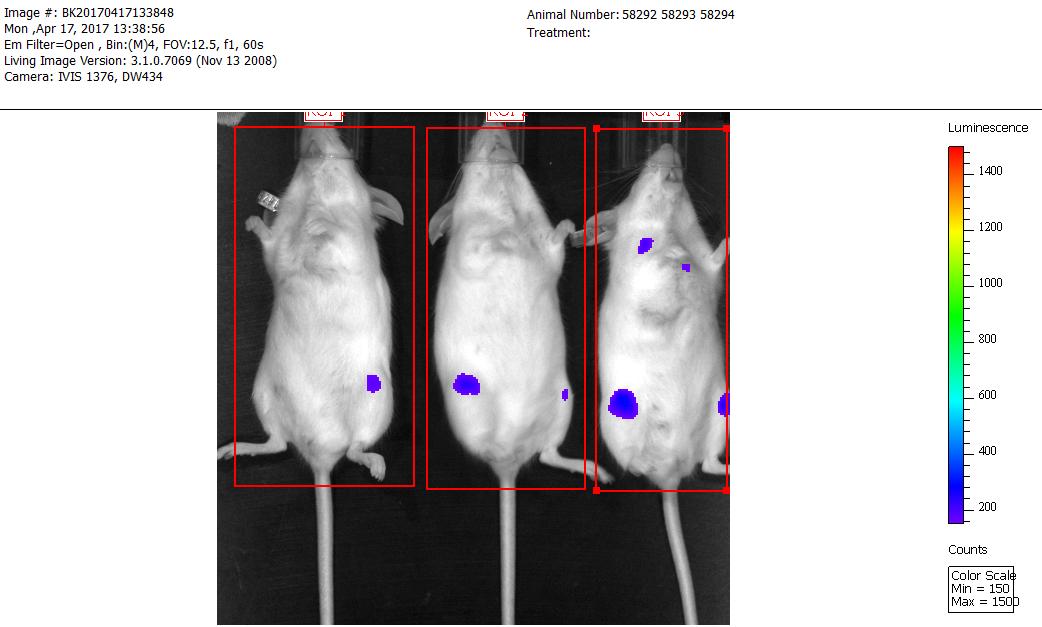

Supplement: Supplementary file 1 — Figure 7G Source Data [file 44318_2026_758_MOESM1_ESM.zip › wt/wt therapy 1,2,3 d7.jpg]

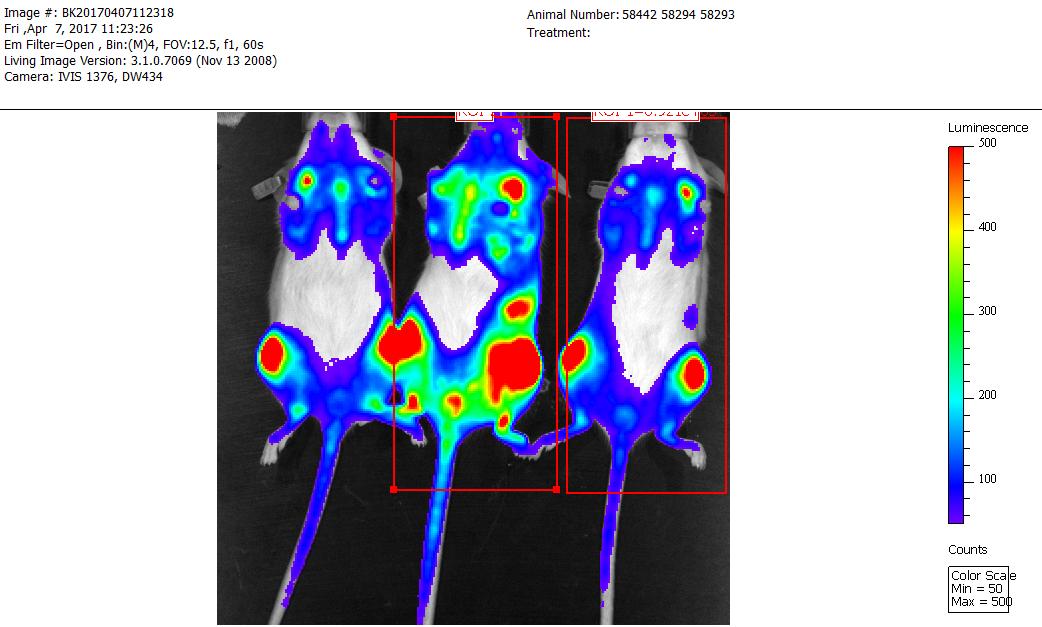

Supplement: Supplementary file 1 — Figure 7G Source Data [file 44318_2026_758_MOESM1_ESM.zip › wt/wt therapy 2 and 3 day 0.jpg]

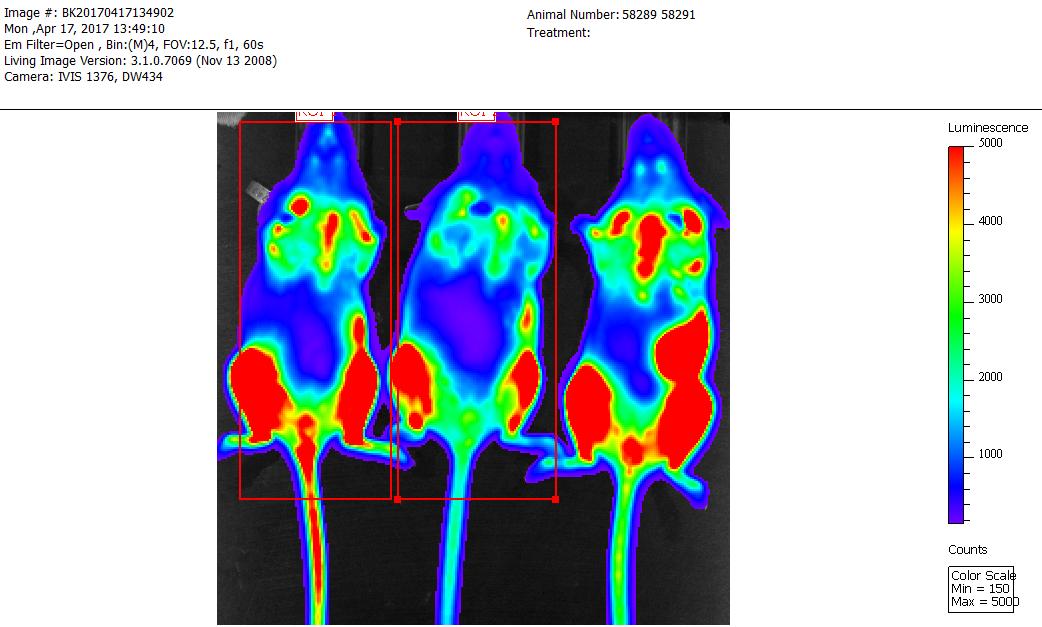

Supplement: Supplementary file 1 — Figure 7G Source Data [file 44318_2026_758_MOESM1_ESM.zip › wt/wt untr 1 and 2 d7.jpg]

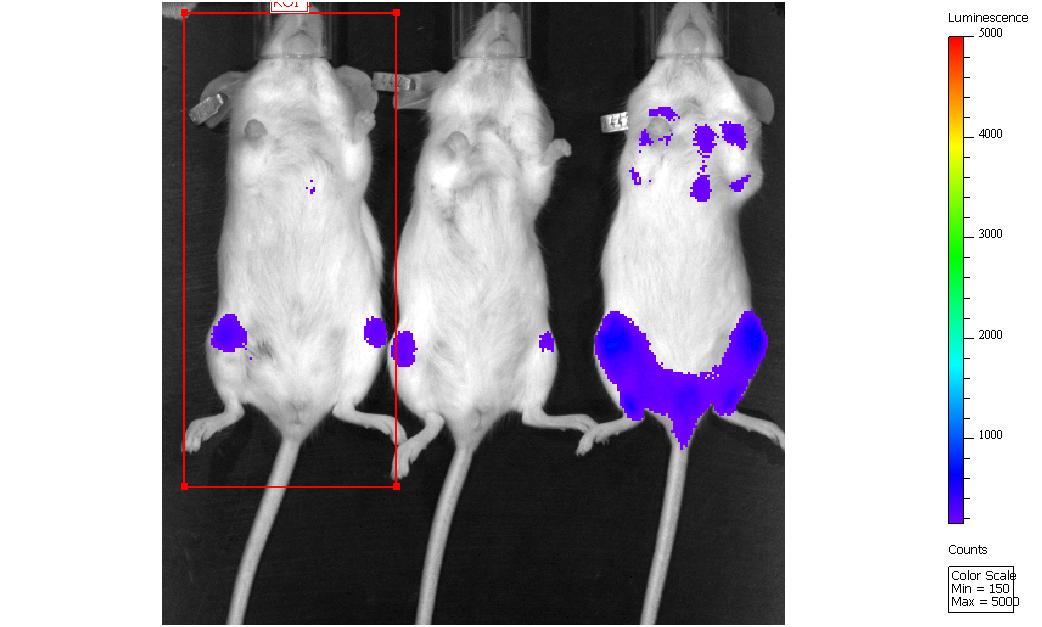

Supplement: Supplementary file 1 — Figure 7G Source Data [file 44318_2026_758_MOESM1_ESM.zip › wt/wt therapy 4 d7.jpg]

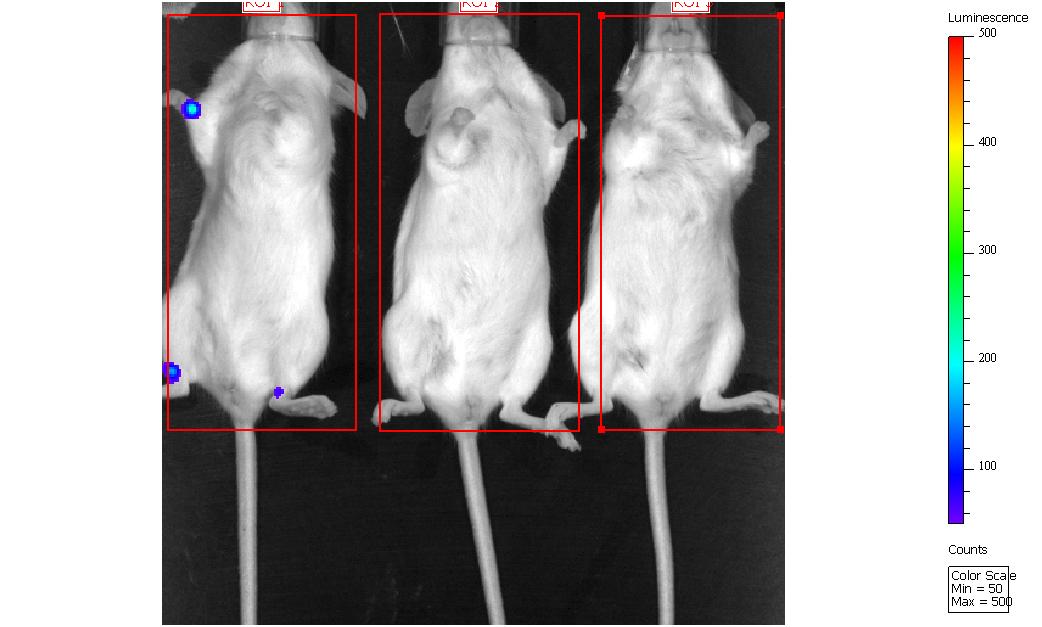

Supplement: Supplementary file 1 — Figure 7G Source Data [file 44318_2026_758_MOESM1_ESM.zip › wt/wt therapy 1,2,3 d 14.jpg]

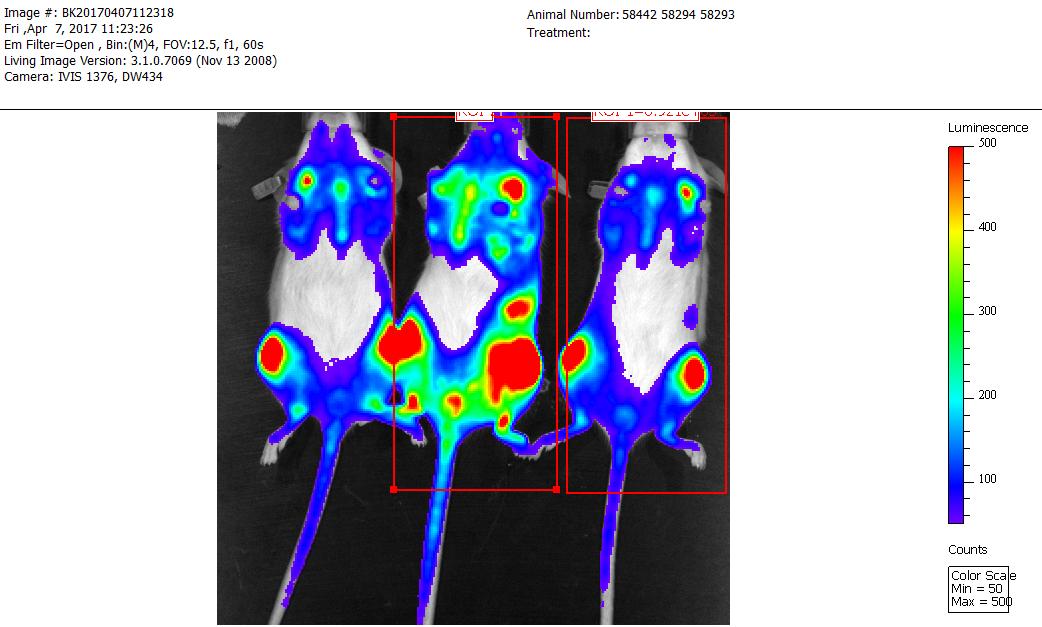

Supplement: Supplementary file 1 — Figure 7G Source Data [file 44318_2026_758_MOESM1_ESM.zip › wt/wt therapy 2 and 3 day 0 - Kopie.jpg]

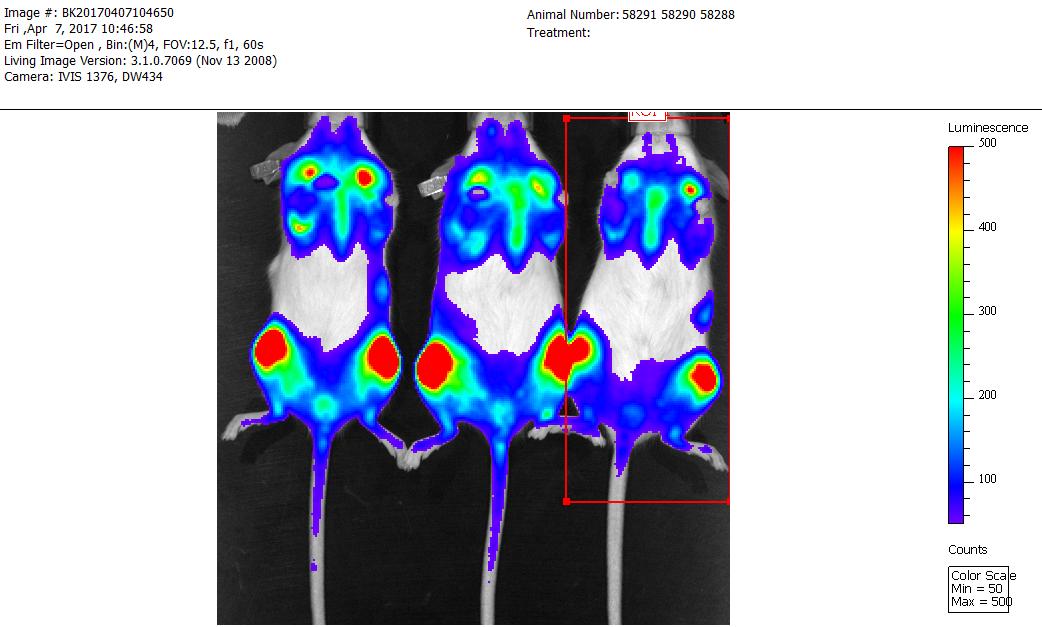

Supplement: Supplementary file 1 — Figure 7G Source Data [file 44318_2026_758_MOESM1_ESM.zip › wt/wt therapy 4 d0.jpg]
